# Mixing Rates of the Geometrical Neutral Lorenz Model

**DOI:** 10.1007/s10955-023-03212-5

**Published:** 2023-12-08

**Authors:** Henk Bruin, Hector Homero Canales Farías

**Affiliations:** Faculty of Mathematics, University of Vienna, Vienna, Austria

**Keywords:** Polynomial decay of correlations, Neutral geometrical Lorenz flow, Mixing, Neutral fixed point, Primary 37D25, Secondary 37C10

## Abstract

The aim of this paper is to obtain polynomial decay of correlations of a Lorenz-like flow where the hyperbolic saddle at the origin is replaced by a neutral saddle. To do that, we take the construction of the geometrical Lorenz flow and proceed by changing the nature of the saddle fixed point at the origin by a neutral fixed point. This modification is accomplished by changing the linearised vector field in a neighbourhood of the origin for a neutral vector field. This change in the nature of the fixed point will produce polynomial tails for the Dulac times, and combined with methods of Araújo and Melbourne (used to prove exponential mixing for the classical Lorenz flow) this will ultimately lead to polynomial upper bounds of the decay of correlations for the modified flow.

## Introduction

The study of flows on surfaces and higher-dimensional manifolds has caught the interest of many scientists because of its numerous applications such as Hamiltonian flows, geodesic and horocycle flows, billiard flows or flows from meteorological models. These flows are usually equipped with a natural invariant measure $\mu$, for instance the SRB-measure.

The main goal is to have a better understanding of the properties of these flows, such as hyperbolicity, ergodicity, mixing (or at least weak mixing) and, in chaotic settings, rates of mixing; that is, we would like to investigate the asymptotic behaviour of the correlation coefficients

1 $$\begin{aligned} \rho _t(v,w)=\Bigg |\int _M v\cdot w\circ f^td\mu -\int _M vd\mu \int _M wd\mu \Bigg |, \end{aligned}$$

where $f^t : M \rightarrow M$ is a flow acting on a manifold *M* and $\mu$ its SRB-measure, and for observables *v*, *w* chosen from an appropriate Banach space. Knowing the rates of mixing is very helpful for proving other ergodic and statistical properties since mixing is one of the strongest statistical properties.

Obtaining good mixing rates for flows, even for hyperbolic flows, is far more difficult than for maps. Some seminal ideas were provided by Liverani [29] and Dolgopyat [20, 21], with applications of these methods in e.g., [9, 10, 16]. To obtain sharp estimates in the polynomial setting, the operator renewal theory techniques developed by Sarig [34] and Gouëzel [24] are the only ones available.

The model we would like to study is probably one of the most emblematic ones, the Lorenz flow. In the mid seventies Afraĭmovič, Bykov and Shilnikov [1] and independently Guckenheimer and Williams [25] introduced the geometric Lorenz attractor to model the original Lorenz attractor. Our research focuses on a modified version of this geometrical model and study its rate of mixing, based on the precise estimates in [17] of Dulac times associated to a neutral saddle.

Recently, Araújo and Melbourne in [3] proved that the geometrical Lorenz flow (and hence the classical Lorenz flow), also enjoys exponential mixing. It is techniques from their papers, specifically $C^{1+\alpha }$ smoothness of the stable foliation, that leads eventually to the claimed mixing rates.

### The Framework

**Fig. 1:**
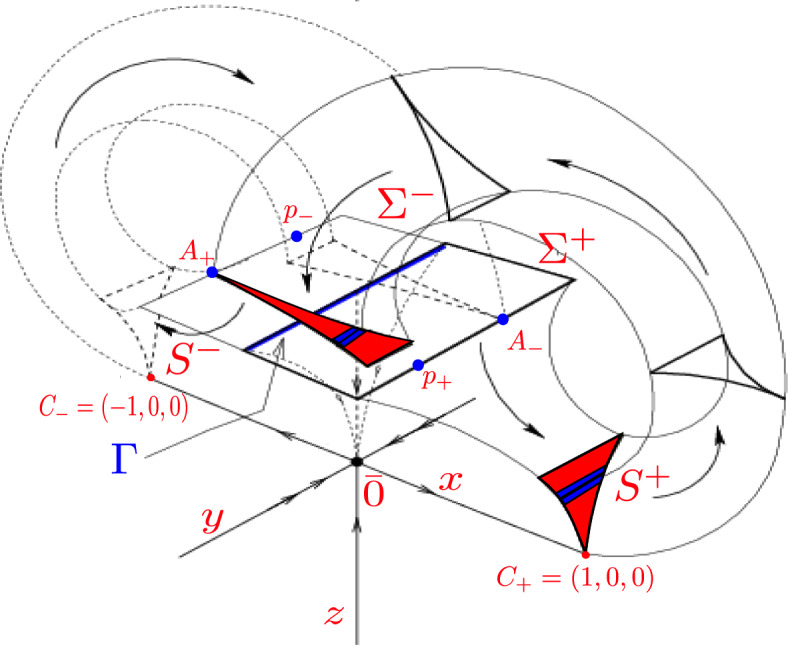
The Guckenheimer-Williams geometric model of the Lorenz flow (Image taken from [4])

The geometrical Lorenz flow can be seen as the natural extension of a suspension semiflow built over a certain type of one-dimensional expanding map $f_{{\text {Lor}}}$. We first consider the cross-section $\Sigma$ transversal to the flow and the Poincaré map $P_{{\text {Lor}}} : \Sigma \rightarrow \Sigma$, which is decomposed in two parts. The first one is the Dulac map, denoted by $P_1$, deals with the local behaviour near the origin and is obtained by considering a linear system in a neighbourhood of the origin; that is, we take the flow ${\text {X}}^t$ obtained from the linear system

2 $$\begin{aligned} {\dot{x}}= & {} \quad \lambda _u x\nonumber \\ {\dot{y}}= & {} \quad -\lambda _s y\nonumber \\ {\dot{z}}= & {} \quad -\lambda _{ss} z \end{aligned}$$

where $\lambda _u$, $\lambda _s$ and $\lambda _{ss}$ denote the unstable, stable and strong stable eigenvalues of the original Lorenz system, respectively. Then we let points in $\Sigma$ flow under ${\text {X}}^t$ until flow time $\tau '_{{\text {Lor}}}:= \min \{ t > 0: {\text {X}}^t \in S\} = -\lambda _{u}^{-1}\ln (|x|)+\mathcal {O}(\ln (|x|))$ as $x \rightarrow 0$. Thus we have that $X^{\tau '_{{\text {Lor}}}}=P_1 : \Sigma \rightarrow S$, where $S^{\pm }$ is the image of $\Sigma ^{\pm }$ under $P_1$ and has a cusp-like shape, see Fig. 1.

The second part, denoted by $P_2$, consists of the return of *S* to $\Sigma$ and simulates the random turns of a regular orbit around the origin and describes a butterfly-like shape. This is done by a composition of a rotation, expansion and translation with hitting time $\tau _2(x)\in C ^{\epsilon }$. Thus, the full return time of the Poincaré map $P_{{\text {Lor}}}=P_2\circ P_1$ is given by

3 $$\begin{aligned} r_{{\text {Lor}}}(x)=\tau '_{{\text {Lor}}}(x)+\tau _2(x). \end{aligned}$$

As we will see later, the lines in the *y*-direction (*i.e.,*, parallel to the *y* axis) in $\Sigma$ form the stable foliation, invariant under $P_{Lor}$; that is, for any leaf $\gamma$ of this foliation, its image $P_{{\text {Lor}}}(\gamma )$ is contained in a leaf of the same foliation. By quotienting out the stable direction we can rewrite the Poincaré map as a skew-product; that is, $P_{{\text {Lor}}}(x,y)=(f_{{\text {Lor}}}(x),g_{{\text {Lor}}}(x,y))$.

The **geometric Lorenz flow** is the couple $(W,X^t_W)$, where $W=\{{\text {X}}^t({\bar{x}})\;|\;{\bar{x}}\in \Sigma ,\;t\in \mathbb {R}^+\}$. Consider $U=\bigcup _{{\bar{x}}\in \Sigma }X_W^{[0,r_{{\text {Lor}}}(x)]}({\bar{x}})$, then the **geometric Lorenz attractor** (of the corresponding vector field) is given by $\Lambda _{{\text {Lor}}}=\bigcap _{t>0} X_W^t(U)$.

In [3], exponential mixing for the geometrical Lorenz flow was proven under two conditions: the stable foliation has to be $C ^{1+\alpha }$ and a uniform non-integrability (UNI) condition needs to be satisfied.

The modified version is obtained by changing the local behaviour near the origin. We achieve this by replacing the linear system for the following system;

4 $$\begin{aligned} \left( \begin{array}{ccc} {\dot{x}}\\ {\dot{y}}\\ {\dot{z}}\\ \end{array}\right) = Z\left( \begin{array}{ccc} x\\ y\\ z\\ \end{array}\right) = \left( \begin{array}{ccc} x(a_0x^2 + a_1y^2 + a_2z^2)\\ -\ell y(1 + c_0x^2 + c_2z^2)\\ -z(b_0x^2 + b_1y^2 + b_2z^2)\\ \end{array}\right) +\mathcal {O}(4), \end{aligned}$$

where $a_0,\;a_1,\;a_2,\;b_0,\;b_1,\;b_2,\;c_0,\;c_2$ and $\ell >0$, $a_2b_0 < 9a_0b_2$, $\Delta :=a_2b_0-a_0b_2\ne 0$ and $\mathcal {O}(4)$ refers to terms of order four or higher, under the condition that they are of the form $x^2\mathcal {O}(2)$ near the *yz*-plane and $z^2\mathcal {O}(2)$ near the *xy*-plane. This system has a polynomial Dulac time (see (8) and Fig. 3) given by;

5 $$\begin{aligned} \tau '_{{\text {Neu}}}:= \min \{ t > 0: {\text {N}}^t \in S\} = |x|^{-\frac{1}{\beta _2}}(1+\mathcal {O}(|x|^{\frac{1}{2\beta _2}})), \end{aligned}$$

as $x\rightarrow 0$ and $\beta _2=\frac{a_2+b_2}{2b_2}$. To obtain the flow time $\tau '_{Neu}$, we make use of the estimates of the Dulac map and the tails of the return map obtained by Bruin and Terhesiu in [17]. This change of flow time, from logarithmic to polynomial, will ultimately allow us to deduce the polynomial decay of correlations.

We denote by ${\text {N}}^t$ the flow obtained from the system given by (4). This change in the local behaviour near the origin leads to a change on the map $P_1$; that is, we have now ${\text {N}}^{\tau '_{{\text {Neu}}}}=D_1 : \Sigma \rightarrow S$. For the second part, the return of *S* to $\Sigma$, we consider the same diffeomorphism $P_2$ with same hitting time. In this way, we obtained the modified Poincaré map $P_{{\text {Neu}}}=P_2\circ d_1$ with return time given by,

6 $$\begin{aligned} r_{{\text {Neu}}}(x)=\tau '_{{\text {Neu}}}(x)+\tau _2(x). \end{aligned}$$

Similarly, we define the **geometric neutral Lorenz flow** as the couple $(W,{\text {N}}^t_W)$, where $W=\{{\text {N}}^t({\bar{x}})\;|\;{\bar{x}}\in \Sigma ,\;t\in \mathbb {R}^+\}$. We consider again $U=\bigcup _{{\bar{x}}\in \Sigma }{\text {N}}^{[0,r_{{\text {Neu}}}(x)]}({\bar{x}})$, the **geometric neutral Lorenz attractor** (of the corresponding vector field) is given by $\Lambda _{{\text {Neu}}}=\bigcap _{t>0}{\text {N}}^t(U)$.

As we will see in more detail in Sect. 2, the geometrical neutral Lorenz flow will be split into three models. **Model 1** is obtained when we take the parameters $c_0=c_2=0$ in (4). **Model 2** when we consider $a_1=b_1=0$. Finally, **Model 3**, the most general, will be given by taking all parameters strictly positive.

### Main Results

Let $C^{\eta }$ be the space of functions that are $\eta$-Hölder in the space direction, and $C^{m,\eta }$ be the space of functions that are $m+\eta$-Hölder (i.e., *m* time differentiable with an $\eta$-Hölder *m*-th derivative) in the flow direction, see Sect. 4 for the precise definitions. The main result in this paper is the following theorem:

#### Theorem 1.1

Let ${\text {N}}^t : \Lambda _{{\text {Neu}}} \rightarrow \Lambda _{{\text {Neu}}}$ be the geometrical neutral Lorenz flow for Model 1 and Model 2 obtained from the neutral form given by (4), with corresponding parameters. $\Lambda _{{\text {Neu}}}$ its attractor and its SRB measure $\mu$. Then $N^t$ has polynomial decay of correlations (with exponent $\beta _2=\frac{a_2+b_2}{2b_2}$); that is, there exist $m\ge 1$ and a constant $C>0$ such that for observables $v\in C^{\eta }(M)\cap C^{0,\eta }(M), w\in C^{m,\eta }(M)$, and time $t>1$ we have

$$\begin{aligned} \rho _t(v,w)\le&C(\left\Vert v\right\Vert _{{C^{\eta }}}+\left\Vert v\right\Vert _{{C^{0,\eta }}})\left\Vert w\right\Vert _{{C^{m,\eta }}} t^{-\beta _2}. \end{aligned}$$

A first question that presents itself is of course if these bounds are sharp, and if current operator renewal theory methods [24, 34] cannot prove that. We say more on this at the end of Sect. 4.

For the proof of Theorem 1.1, we obtain an explicit form of the Poincaré map, since we can solve the differential equation in the *y* component. Thus we are able to prove polynomial decay of correlations by using the results on non-uniformly hyperbolic flows established by Bálint et al. in [12].

For the third model the situation is more subtle since, to our knowledge, finding the solution of the differential equation in the *y* component is next to impossible. To overcome this problem we will analyse and compare, with numerical methods, the limit behaviour of the Dulac maps obtained in [15] and [17] and adapted to our framework. More precisely, we will analyse the limit behaviour of the maps $D_1 : \Sigma \rightarrow S$ obtained for each Neutral model. This is sufficient since the Poincaré maps considered in this work are given by $P_{{\text {Neu}}}=P_2\circ D_1$, where $P_2$ is a diffeomorphism and the map $D_1$ is the Dulac map from the cross-section $\Sigma$ to the cusps *S*, which depends on the differential equation being considered. Therefore, the behavioural changes exhibited by the map $P_{{\text {Neu}}}$ are represented by the changes of the map $D_1$. Dulac in [22] made a significant contribution to solving Hilbert’s 16th problem by incorporating his map as an element to establish that polynomial vector fields in the plane possess a limited number of limit cycles, demonstrating that they cannot have an infinite number of such cycles.

The numerical analysis on the behaviour of the Dulac maps will give us the plausibility of the following conjecture.

#### Conjecture 1.2

Let ${\text {N}}^t : \Lambda _{{\text {Neu}}} \rightarrow \Lambda _{{\text {Neu}}}$ be the geometrical neutral Lorenz flow for Model 3 obtained from the neutral form given by (4), with the corresponding parameters. $\Lambda _{{\text {Neu}}}$ its attractor and its SRB measure $\mu$. Then $N^t$ has polynomial decay of correlations (with exponent $\beta _2=\frac{a_2+b_2}{2b_2}$).

The organization of this paper is as follows: In Sect. 2 we will give the construction of the Poincaré maps of the Neutral Model 1 and 2. In Sect. 3 we will be devoted to the proof that the stable foliation for the geometrical neutral models is $C ^{1+\alpha }$ and the UNI condition is satisfied by adapting the existing proofs for the geometrical Lorenz model. Section 4 contains the framework of non-uniformly hyperbolic flows and the proof of Theorem 1.1. Finally, in Sect. 5 we will present the numerical analysis and results we obtained for the Dulac map and the tails of the return map.

For the remaining of this paper we will adopt the following notation.

#### Notation 1.3

In order to avoid excessive notation of the higher order terms, obtained from the estimates of the Dulac time given in [17], we will write $A_1(x,\beta )$ and $A_2(x,\beta _2)$ to denote $\xi |x|^\beta (1+\mathcal {O}(|x|^{\frac{1}{2\beta _2}}))$ and $\zeta |x|^{-\frac{1}{\beta _2}}(1+\mathcal {O}(|x|^{\frac{1}{2\beta _2}}))$, respectively, where $\beta _0=\frac{a_0+b_0}{2a_0}$, $\beta _2=\frac{a_2+b_2}{2b_2}$, $\beta =\frac{\beta _0}{\beta _2}$, $\xi$ and $\zeta$ are constants given in [17], namely in Theorem 1.1 and the proof of Proposition 2.1. $X\in \mathfrak {X}^{r}(M)$ will denote the vector space of $C^r$ vector fields in a manifold *M* with the $C^r$ topology.

## The Poincaré Maps

### Neutral Model 1

To create the modified models we will apply local surgery in a neighbourhood of the hyperbolic saddle equilibrium of the geometrical Lorenz model, namely the origin, and transform it into a neutral equilibrium. We do this because we aim to slow down the orbit and thus increase the time that orbits take to flow from the cross-section $\Sigma$ to the cusps *S*, see Fig. 1, and see the changes this new motion produces in the decay of correlations. The flow obtained from this modification will be an almost Anosov flow [27, 28]. Existence of a finite or infinite SRB measure for two-dimensional almost Anosov diffeomorphisms was already proven in [27, 28]. Bruin and Terhesiu in [17] proved mixing rates in the infinite SRB measure setting for almost Anosov diffeomorphism and established the required spectral properties for the transfer operator (acting on an appropriate anisotropic Banach space of distributions) of an induced map so as to obtain optimal rates of mixing. Furthermore, they gave more precise tail estimates for the inducing scheme. We will take advantage of these methods and estimates and use them to deduce the rates of mixing of our almost Anosov flow.

**Fig. 2:**
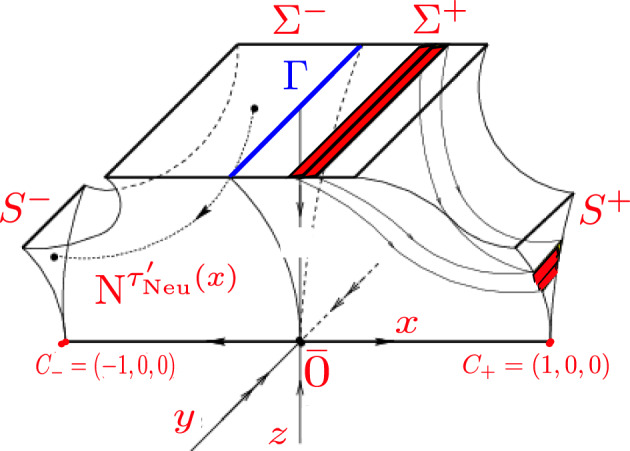
The map $D_1$ (Image taken from [4])

We consider now $\Sigma ^{*}=\{(x,y,1)\in \mathbb {R}^3\;|\;|x|\le 1,|y|\le 1\}$, $\Sigma ^{-}=\{(x,y,1)\in \Sigma ^{*}\;|\;x<0\}$, $\Sigma ^{+}=\{(x,y,1)\in \Sigma ^{*}\;|\;x>0\}$, $\Sigma =\Sigma ^{+}\cup \Sigma ^{-}=\Sigma ^{*}\setminus {\tilde{\Gamma }}$, where ${\tilde{\Gamma }}=\{(x,y,1)\in \Sigma ^{*}\;|\;x=0,\}$ and $S=S^{+}\cup S^{-}$ where $S^{\pm }$ is the image of $\Sigma ^{\pm }$ under the Dulac map $D_1$, see Fig. 2. The section $\Sigma$ is transversal to the flow and every trajectory eventually crosses $\Sigma$ in the direction of the negative axis *z*. Then for each $(x,y,1)\in \Sigma$, the time $\tau '_{{\text {Neu}}}$ such that ${\text {N}}^{\tau '_{{\text {Neu}}}}(x,y,1)\in S$ is determined by the estimates of the Dulac map provided in [17], as we will explain now.

Let us start with the Neutral model 1; that is, we consider a neighbourhood *U* of the fixed point ${{\bar{0}}} = (0,0,0)$ where the vector field has the form (4) (in local Euclidean coordinates) with $c_0=c_2=0$ and the other parameters satisfying the constraints given before. Note that *U* is taken much smaller than the scale of Fig. 2. This vector field, denoted by *Z*, is cubic at ${{\bar{0}}}$ in the direction transversal to the stable manifold of ${{\bar{0}}}$, but this is the only source of non-hyperbolicity. The *y*-axis is invariant and all solutions tend to ${{\bar{0}}}$. The divergence is given by

$$\begin{aligned} {\text {Div}}(Z)=(3a_0-b_0)x^2+(a_1-b_1)y^2+(a_2-3b_2)z^2-\ell . \end{aligned}$$

Since we want the flow to shrink volume exponentially fast, as does the Lorenz flow, we need ${\text {Div}}(Z) \le -c<0$. Therefore, we let $\ell$ be large enough such that $(3a_0-b_0)x^2+(a_1-b_1)y^2+(a_2-3b_2)z^2<\ell$ for all $(x,y,z) \in U$. The solution for the *y*-component is given by $y(t)=y_0{\text {e}}^{-\ell t}$. Thus we obtain a non-autonomous system of differential equations, since the contribution of the *y*-component to the *x* and *z*- component is exponentially small as time increases, these terms are of smaller order than the higher order terms. Thus we are left with the two-dimensional system studied in [17]:

7 $$\begin{aligned} \left( \begin{array}{ccc} {\dot{x}}\\ {\dot{z}}\\ \end{array}\right) = Z_{{\text {hor}}}\left( \begin{array}{ccc} x\\ z\\ \end{array}\right) = \left( \begin{array}{ccc} x(a_0x^2+a_2z^2)\\ -z(b_0x^2+b_2z^2)\\ \end{array}\right) . \end{aligned}$$

Now let ${\mathcal {W}}^s$ and ${\mathcal {W}}^u$ be two mutually tranversal foliations of the interior of $U \cap \{\text {positive quadrant}\}$ that is invariant under the flow of (7) and such that

- the leaves of ${\mathcal {W}}^s$ accumulate in $C^1$ topology on the stable manifold of (0, 0) and are transversal to the unstable manifold of (0, 0), and
- the leaves of ${\mathcal {W}}^u$ accumulate in $C^1$ topology on the unstable manifold of (0, 0) and are transversal to the stable manifold of (0, 0).

One would like to use the stable and unstable foliation of the horizontal flow $\phi _{hor}$ of (7) for ${\mathcal {W}}^s$ and ${\mathcal {W}}^u$, but as long as we defined the flow only locally, the above properties suffice.

**Fig. 3:**
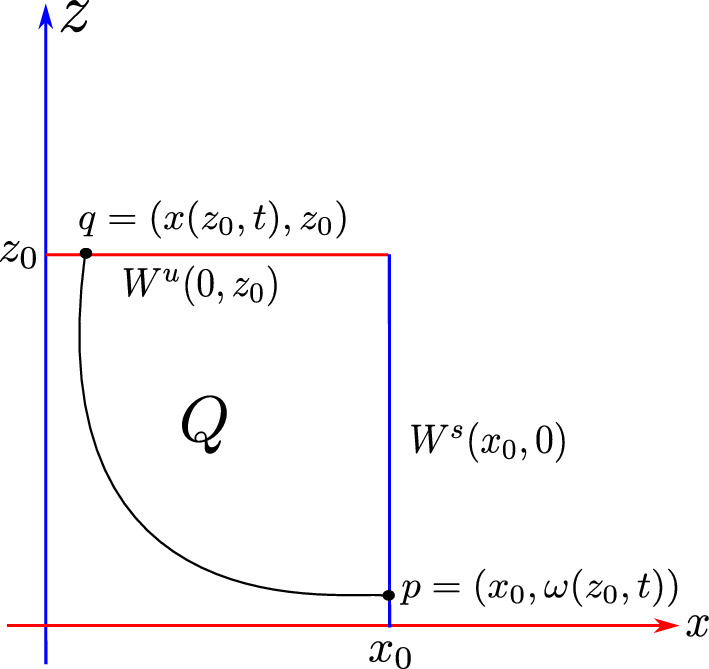
The Dulac map $D : W^u(0,z_0) \rightarrow W^s(z_0,0)$

Now fix an unstable leaf $W^u(0,z_0) \in {\mathcal {W}}^s$ and a stable leaf $W^s(x_0,0) \in {\mathcal {W}}^u$, then the Dulac map $D : W^u(0,z_0) \rightarrow W^s(x_0,0)$, shown in Fig. 3, assigns the first intersection $\phi ^T(x,z_0)$ of the integral curve through $(x,z_0)$ with the stable leaf $W^s(x_0,0)$, where $x\in W^u(0,z_0)$, $\phi ^t(x,z)$ is the flow from (7) and *T* is the exit time. The estimates for the map *D* and the flow time given in [17] are:

8 $$\begin{aligned} \omega =D(x)=c(z_0)x^\beta (1+\mathcal {O}(x^{\frac{1}{2\beta _2}})) \end{aligned}$$

and

9 $$\begin{aligned} \tau '_{{\text {Neu}}}(x)=A_2(x,\beta _2), \end{aligned}$$

where $\beta =\frac{\beta _0}{\beta _2}$ for $\beta _0=\frac{a_0+b_0}{2a_0}$ and $\beta _2=\frac{a_2+b_2}{2b_2}$.

More relevant to the proof of the decay of correlations of the neutral geometrical Lorenz flow is the estimate of the tails of the return map which we state in the following theorem.

#### Theorem 2.1

[17, Theorem 1.1] Let *Q* be a region bounded by the stable and unstable manifolds of 0 and a leaf $W^s(x_0,0) \in {\mathcal {W}}^s$ and a leaf $W^u(0,z_0) \in {\mathcal {W}}^u$, see Fig. 3. Let $\beta ^{*}=\frac{1}{2}\min \{1,\frac{a_2}{b_2},\frac{b_0}{a_0}\}$, then there exists $C_0>0$ a constant such that $\mu (\varphi >n)=C_0n^{-\beta _2}(1+\mathcal {O}(n^{-\beta ^{*}}))$, where $\varphi =\inf \{t > 0\;|\; \phi _{hor}^t(z)\notin Q\}$ and $\mu$ is Lebesgue measure^1^ on $W^u(0,z_0)$.

Putting all together we get the following expression for the map $D_1$

10 $$\begin{aligned} D_1(x,y,1)&={\text {N}}^{\tau '_{{\text {Neu}}}(x)}(x,y,1) \nonumber \\&=\Big (1,y{\text {e}}^{-\ell A_2(x,\beta _2)},A_1(x,\beta )\Big ), \end{aligned}$$

where the functions $A_2(x,\beta _2 )$ and $A_1(x,\beta )$ come from Notation 1.3.

We make the following observations:

#### Observation 2.2

1. $D_1(\Sigma ^{\pm })$ has the shape of a cusp at $(\pm 1,0,0)$ and (with some abuse of notation) we will denote these images as $S^{\pm }$ and $S=S^+\cup S^-$.
2. Denote by $\ell _v(c)=\{(x,y,1)\in \Sigma \;|\;x=c\}$, where *c* is a constant, the line segments in $\Sigma$ parallel to the *y*-axis and by $\ell _h(c)=\{(\pm 1,y,z)\in S\;|\;z=c\}$, the line segments in *S* parallel to the *y*-axis. Then $D_1(\ell _v(c_0))=\ell _h(c_1)$; that is, the map $D_1$ takes the *y*-direction lines in $\Sigma$ to the horizontal line segments in *S* as illustrated in Fig. 2.

**Fig. 4:**
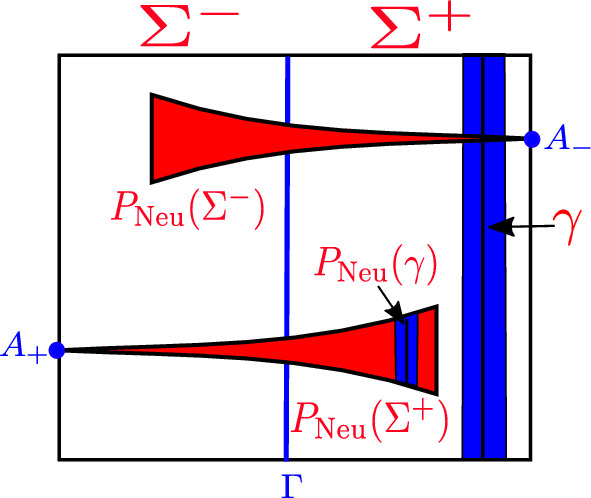
The Poincaré map for the neutral geometrical model

The return of the cusps *S* to the cross-section $\Sigma$ is described by the map $P_2=T\circ E_a\circ R_{\theta }$, where $R_\theta$ is a rotation by an angle of $\theta =\frac{3\pi }{2}$ and the rotation axis are the boundaries of the cross-section $\Sigma$ parallel to the *y*-axis, $E_a$ is an expansion by a factor of $a>1$ in the *x*-direction and a translation *T* such that the unstable direction which starts from the origin is sent to the boundary of $\Sigma$; that is, we want to send the cusp points $C_{\pm }$ to $A_{\pm }$, see Fig. 1. Thus, the full Poincaré map $P_{{\text {Neu}}}=P_2\circ D_1 : \Sigma \rightarrow \Sigma$, shown in Fig. 4, is given by,

11 $$\begin{aligned} P_{{\text {Neu}}}(x,y) = {\left\{ \begin{array}{ll} \left( aA_1(x,\beta )-1,\;y{\text {e}}^{-\ell A_2(x,\beta _2)}-\dfrac{1}{2} \right) , &{} \text {if} \; x \in (0, 1];\\ \left( aA_1(x,\beta )+1,\;y{\text {e}}^{-\ell A_2(x,\beta _2)}+\dfrac{1}{2}\right) , &{} \text {if} \; x \in [-1, 0).\\ \end{array}\right. } \end{aligned}$$

In the positive quadrant the matrix $DP_{{\text {Neu}}}$ has eigenvalues $\lambda _1=a\frac{\beta _0}{\beta _2} |x|^{\frac{\beta _0}{\beta _2}-1}$ and $\lambda _2={\text {e}}^{-\ell A_2(x,\beta _2)}$. By restricting $\frac{1}{2}<\beta _0<2$ we have that $0<\dfrac{\beta _0}{\beta _2}<1$ since $\beta _2>2$. Then $\lambda _1\rightarrow \infty$ as $x\rightarrow 0$. For the other eigenvalue we have that $\lambda _2<1$ and $\lambda _2\rightarrow 0$ as $x \rightarrow 0$ since $A_2(x,\beta _2)\rightarrow \infty$ as $x \rightarrow 0$. Thus the modified Poincaré map $P_{{\text {Neu}}}$ is hyperbolic when *x* approaches the origin.

The foliation given by the lines $\ell _v(c)$ is invariant under the map $P_{{\text {Neu}}}$; that is, given any leaf $\gamma$ of this foliation its image $P_{{\text {Neu}}}(\gamma )$ is contained in a leaf of the same foliation (See Fig. 4). Therefore, we can express $P_{{\text {Neu}}}$ as $P_{{\text {Neu}}}(x,y)=(f_{{\text {Neu}}}(x),g_{{\text {Neu}}}(x,y))$, where $f_{{\text {Neu}}} : I\setminus \{0\} \rightarrow I$ is a Lorenz-like map with exponent $\beta =\frac{\beta _0}{\beta _2}$ with $\beta _0=\frac{a_0+b_0}{2a_0}$ and $\beta _2=\frac{a_2+b_2}{2b_2}$; that is, $f_{{\text {Neu}}}$ is given by,

12 $$\begin{aligned} f_{{\text {Neu}}}(x)= {\left\{ \begin{array}{ll} aA_1(x,\beta )-1, &{} \text {if} \; x \in (0, 1];\\ 1-a|A_2(x,\beta )|, &{} \text {if} \; x \in [-1, 0),\\ \end{array}\right. } \end{aligned}$$

and the function $g_{{\text {Neu}}} : (I{\setminus }\{0\})\times I \rightarrow I$, where $I=[-1,1]$, satisfies the following:

1. The map $$g_{{\text {Neu}}}$$ is piecewise $$ C ^{2}$$ and for fixed $$x_0$$, the map $$g_{{\text {Neu}}}(x_0,y)$$ is a contraction in the *y*-direction, i.e.,
  $$\begin{aligned} d(g_{{\text {Neu}}}(x_0,y_1),g_{{\text {Neu}}}(x_0,y_2))\le cd(y_1,y_2), \end{aligned}$$
  where *d* is the Euclidean distance in *I* and $0<c<1$.
2. $DP_{{\text {Neu}}}$ has the following bound on its partial derivatives:
  - For all $(x,y)\in \Sigma$ we have $\partial _yg_{{\text {Neu}}}(x,y)={\text {e}}^{-\ell A_2(x,\beta _2)}$. Since $\beta _2>2$ and $|x|\le 1$, there is $0<\eta <1$ such that
    $$\begin{aligned} |\partial _yg_{{\text {Neu}}}(x,y)|<\eta . \end{aligned}$$
  - For $(x,y)\in \Sigma$ with $x\ne 0$ we have $\partial _xg_{{\text {Neu}}}(x,y)=\frac{\ell }{\beta _2}y(A_2(x,\beta _2))'$ ${\text {e}}^{-\ell A_2(x,\beta _2)}$. Since $1<1+\dfrac{1}{\beta _2}<\dfrac{3}{2}$ and $|y|,\;|x|\le 1$ we get that $|\partial _xg_{{\text {Neu}}}(x,y)|$ is bounded. In fact, it tends to zero exponentially fast as *x* approaches the origin.
3. From g2.a) above follows the uniform contraction of the foliation given by the lines $\ell _v(c)$; in other words, there is $C>0$ such that, for any given leaf $\gamma$ of the foliation and for $y_1,\; y_2\in \gamma$, we have
  $$\begin{aligned} d(P_{{\text {Neu}}}^n(y_1),P_{{\text {Neu}}}^n(y_2))\le C\eta ^nd(y_1,y_2), \end{aligned}$$
  when $$n\rightarrow \infty $$.

### Neutral Model 2

Now we will consider the Neutral model 2; that is, we consider the same neighbourhood *U* of the origin where the flow has the local form given by (4) with $a_1=b_1=0$ and the remaining parameters satisfying the same constraints stated in this framework. Again ${{\bar{0}}}=(0,0,0)$ is the only neutral periodic orbit and the vector field is cubic in the direction transversal the stable manifold of ${{\bar{0}}}$, but this is the only source of non-hyperbolicity. If $x=0$ and $z=0$, then we see that ${\dot{x}}=0$, ${\dot{y}}=-\ell y$ and ${\dot{z}}=0$. Hence, the *y*-axis is invariant and all solutions tend to the origin as in the previous model. Moreover, since ${\dot{x}}$ and ${\dot{z}}$ are decoupled from *y*, we have (7). Thus the asymptotics for the Dulac map and the flow time given in [17] follow. Also ${\text {Div}}(Z)=(3a_0-\ell c_1-b_0)x^2+(a_2-\ell c_2-3b_2)z^2-\ell$. Since we want a flow that shrinks volume exponentially fast as before, we take $\ell$ large enough so that $\frac{(3a_0-b_0)x^2+(a_2-3b_2)z^2}{1+c_0x^2+c_2z^2}<\ell$ for all $(x,y,z) \in U$.

We consider the same cross-section $\Sigma$ as before and proceed to construct the Poincaré map in the same way. Denote by $N^t$ the flow obtained from (4) with the pertinent constraints in the parameters; that is, $N^t(x,y,z)$ $=(x(t),y(t),z(t))$. By (8) we obtain the following form for the flow,

13 $$\begin{aligned} N^{t(x,z)}(x,y,z)=(x_0,y(t(x,z)),\omega (z,t(x,z)). \end{aligned}$$

Note that ${\dot{y}}=y(-\ell (1+c_0x^2+c_2z^2))$, applying Grönwall’s Lemma we get,

14 $$\begin{aligned} y(t)&=y_0\exp (-\ell \int _0^t(1+c_0x^2+c_2z^2)ds)\nonumber \\&=y_0{\text {e}}^{-\ell t}\exp (-\ell \int _0^t(c_0x^2+c_2z^2)ds). \end{aligned}$$

By the estimates of the Dulac map and since $z\in \Sigma$, we obtain that the time *t*(*x*, *z*) becomes a function of the variable *x* and the integral $\int _0^t(c_0x^2+c_2z^2)ds$ can be expressed as a function *q* of the variable *x*. Observe that $q(x)>0$ for every *x*. Therefore we get that,

15 $$\begin{aligned} y(t)&=y_0{\text {e}}^{-\ell t}{\text {e}}^{-\ell q(x)}. \end{aligned}$$

Hence *y*(*t*) decreases exponentially fast as before but with a faster rate. All together, we get that the map $D_1 : \Sigma \rightarrow S$ is given by

16 $$\begin{aligned} D_1(x,y,1)&={\text {N}}^{\tau '_{{\text {Neu}}}(x)}(x,y,1) \nonumber \\&=\Big (1,y{\text {e}}^{-\ell (A_2(x,\beta _2)+q(x))},A_1(x,\beta )) \Big ), \end{aligned}$$

where $\beta =\frac{\beta _0}{\beta _2}$, compare this with (10). The statements from Observation 2.2 for this new version of the map $D_1$ will also hold. To finish the construction of the Poincaré map we compose now with the map $P_2$. Therefore, the full return map $P_{{\text {Neu}}} : \Sigma \rightarrow \Sigma$ of $\Sigma$ is given by

17 $$\begin{aligned} P_{{\text {Neu}}}(x,y) = {\left\{ \begin{array}{ll} \left( aA_1(x,\beta )-1,\;y{\text {e}}^{-\ell (A_2(x,\beta _2)+q(x))}-\dfrac{1}{2}\right) , &{} \text {if} \; x \in (0, 1];\\ \left( 1-a|A_1(x,\beta )|,\;y{\text {e}}^{-\ell (A_2(x,\beta _2)+q(x))}+\dfrac{1}{2}\right) , &{} \text {if} \; x \in [-1, 0),\\ \end{array}\right. } \end{aligned}$$

where $$\beta =\frac{\beta _0}{\beta _2}\in (0,1)$$.

The matrix $DP_{{\text {Neu}}}$ has eigenvalues $\lambda _1=a\beta |x|^{\beta -1}$ and $\lambda _2={\text {e}}^{-\ell (A_2(x,\beta _2)+q(x))}$. Since $\beta \in (0,1)$ we have that $\lambda _1\rightarrow \infty$ as $x\rightarrow 0$. For the other eigenvalue we have that $\lambda _2<1$ and $\lambda _2\rightarrow 0$ as $x \rightarrow 0$ since $(A_2(x,\beta _2)+q(x))\rightarrow \infty$ as $x\rightarrow 0$. Thus the modified Poincaré map $P_{{\text {Neu}}}$ is hyperbolic when *x* approaches the origin.

The properties stated before remain true for this new modified return map $P_{{\text {Neu}}}$ like the invariance of the stable foliation given by the vertical lines $\ell _v(c)$ under the map $P_{{\text {Neu}}}$. Hence, we can express again $P_{{\text {Neu}}}$ as $P_{{\text {Neu}}}(x,y)=(f_{{\text {Neu}}}(x),g_{{\text {Neu}}}(x,y))$, where $f_{{\text {Neu}}} : I\setminus \{0\} \rightarrow I$ is again a Lorenz-like map with exponent $\beta$ (see (12)) and $g_{{\text {Neu}}} : (I\setminus \{0\})\times I \rightarrow I$ satisfy the same properties stated in the previous section. The existence of a unique a.c.i.p and statistical properties such as exponential decay of correlations for observables with bounded variation for the Lorenz-like map $f_{{\text {Neu}}}$ are well established, see for example [35].

## The Stable Foliation and the UNI Condition

### Existence and Regularity of the Strong Stable Foliation

In this subsection we will study the properties of the strong stable foliation $\mathcal {F}^{ss}$ for the neutral geometrical Lorenz model we built in Sect. 2.

For the neutral geometrical Lorenz attractor, denoted by $\Lambda _{{\text {Neu}}}$, we consider the Lorenz attractor $\Lambda _{{\text {Lor}}}$ in an open neighbourhood *U* of the origin. Instead of considering the linearised vector field we consider the vector field given by (4) with the parameters corresponding for model 1 and 2 described in Sect. 2.1 and 2.2, respectively. More precisely, we take an open neighbourhood *U* in which the cross-section $\Sigma$ is contained. Then the Dulac map from $\Sigma$ to *S* has the form given by (10) and (16) for the models 1 and 2, respectively.

This modification yields a different flow time from the cross-section $\Sigma$ to *S*. In the original Lorenz construction we have a logarithmic Poincaré return time but for these modifications we have a polynomial Poincaré return time given by (5). The rest of the construction, however, remains unchanged; that is, the flow constructed from *S* to $\Sigma$ is made by a composition of an expansion, a rotation and a translation. Therefore we have the same hitting time $\tau _2(x)$ and thus the full return time for the modified Poincaré map $P_{{\text {Neu}}}$ is given in (6). The modified Poincaré map $P_{{\text {Neu}}} : \Sigma \rightarrow \Sigma$ is given by (11) and (17) for the model 1 and 2, respectively. We saw that the lines in the *y*-direction, denoted by $\ell _v(c)$, in the cross-section $\Sigma$ form the stable foliation which is preserved by the return map $P_{{\text {Neu}}}$. Thus by quotienting out the stable direction we can rewrite the Poincaré map as a skew-product; that is, $P_{{\text {Neu}}}(x,y)=(f_{{\text {Neu}}}(x),g_{{\text {Neu}}}(x,y))$, where $f_{{\text {Neu}}}$ is a one-dimensional Lorenz-like map.

#### Lemma 3.1

If $a_2b_0 < 9a_0b_2$, then the eigenvalues of $DZ_{(x,y,z)}$ satisfy $0< -\lambda _s< \lambda _u < -\lambda _{ss}$ for all $(x,y,z) \in U$.

#### Proof

The derivative matrix $DZ_{(x,y,z)}$ of the vector field *Z* is

$$\begin{aligned} \begin{pmatrix} 3a_0x^2+a_1y^2+a_0z^2 &{} 2a_1xy &{} 2a_2xz \\ -2c_0\ell xy &{} -\ell (1+c_0x^2+c_2y^2) &{} -2c_2 \ell yz \\ -2b_0xz &{} -2b_1yz &{} -(b_0x^2+b_1y^2+3b_2z^2) \end{pmatrix}. \end{aligned}$$

In finding the eigenvalues, we get $\lambda _{ss} = -\ell$ for Model 1 (i.e., $c_0=c_2=0$) and $\lambda _{ss} = -\ell (1+c_0x^2+c_2z^2)$ for Model 2 (i.e., $a_1=b_1=0$), In both cases, the other two eigenvalues are $\frac{1}{2}\left( {\text {trace}}(A) \pm \sqrt{{\text {trace}}(A)^2 - 4 \det (A)}\right)$ for the submatrix

$$\begin{aligned} A = \begin{pmatrix} 3a_0x^2+a_1y^2+a_0z^2 &{} 2a_2xz \\ -2b_0xz &{} -(b_0x^2+b_1y^2+3b_2z^2) \end{pmatrix}. \end{aligned}$$

To ensure that these eigenvalues are real and of opposite sign, it suffices to check that

$$\begin{aligned} \lambda _u \lambda _s = \det (A) = -(3a_0x^2+a_1y^2+a_0z^2)(b_0x^2+b_1y^2+3b_2z^2)+4a_2b_0x^2z^2 < 0. \end{aligned}$$

The worst case is when $y = 0$, so we consider the terms not including *y* (and divide by 3 for simplicity):

$$\begin{aligned} (a_2b_0-3a_0b_2)x^2z^2 - a_0b_0 x^4 - a_2b_2 z^2 < 0. \end{aligned}$$

Divide by $z^4$ and introduce the new coordinate $u = x^2/z^2$:

18 $$\begin{aligned} -Pu^2 + Qu -R < 0 \quad \text { for } \quad P = a_0b_0, Q = a_2b_0-3a_0b_2, R = a_2b_2. \end{aligned}$$

The left hand side is indeed negative for $u = 0$, and it is negative for all *u* if the equation $-Pu^2 + Qu -R = 0$ has no real solution, i.e., if the discriminant is negative:

$$\begin{aligned} 0 > Q^2 - 4PR = (a_2b_0-3a_0b_2)^2 - 4a_0b_2a_2b_0 = 9a_0^2b_2^2 + a_2^2b_0^2 - 10 a_0b_2a_2b_0. \end{aligned}$$

Divide by $a_2b_0$ and use the new coordinate $\gamma = \frac{a_0b_2}{a_2b_0}$. Then we get the inequality

$$\begin{aligned} 0 > 9\gamma ^2 - 10\gamma + 1 =\left( 3\gamma - \frac{5}{3}\right) ^2 - \frac{16}{9}, \end{aligned}$$

which is equivalent to $|\gamma - \frac{5}{9}| < \frac{4}{9}$. That is, it fails if $\gamma \le \frac{1}{9}$ or $\gamma \ge 1$. Now consider equality in(18) and we divide by $a_2b_0$, which brings it to the form

$$\begin{aligned} -\frac{a_0}{a_2} u^2 + (1-3\gamma ) u - \frac{b_2}{b_0} = 0, \end{aligned}$$

with solutions

$$\begin{aligned} u = \frac{a_2}{2a_0} \left( 1-3\gamma \mp \sqrt{ (1-3\gamma )^2 - 4\gamma } \right) = \frac{a_2}{2a_0} \left( 1-3\gamma \mp \sqrt{ 9 \gamma ^2 - 10 \gamma +1} \right) . \end{aligned}$$

If $\gamma \ge 1$, then these solutions are negative, and since $u = x^2/z^2$, this means that there are no solutions $(x,y,z) \in U$. The remaining case $\gamma \le \frac{1}{9}$ is exactly the excluded case in the lemma. This concludes the proof. $\square$

Using our assumption $a_2b_0 < 9a_0b_2$ and Lemma 3.1, we obtain that the origin is the only point where we have $\lambda _{ss}=-\ell$ and $\lambda _s=\lambda _u=0$. Before continuing, we recall the definitions of a partially hyperbolic set and strongly dissipativity.

#### Definition 3.2

Let $\Lambda$ be a compact invariant set of $X\in \mathfrak {X}^{r}(M), c>0$ and $0<\lambda <1$. We say that $\Lambda$ has a $(c,\lambda )-$**dominated splitting** if the tangent bundle $T_{\Lambda }M$ has a $DX^t$-invariant splitting of sub-bundles

$$\begin{aligned} T_{\Lambda }M=E^1\oplus E^2, \end{aligned}$$

such that for all $t>0$ and $x\in \Lambda$, we have

19 $$\begin{aligned} \left\Vert DX^t|_{E_x^1}\right\Vert _{}\cdot \left\Vert DX^{-t}|_{E^2_{X^t(x)}}\right\Vert _{}<c\cdot \lambda ^t. \end{aligned}$$

We say that $\Lambda$ is **partially hyperbolic** if it has a $(c,\lambda )-$dominated splitting such that $E^1$ is uniformly contracting; that is, for some $c>0$ and all $t>0$ and every $x\in \Lambda$ it holds

20 $$\begin{aligned} \left\Vert DX^t|_{E_x^1}\right\Vert _{}<c\cdot \lambda ^t. \end{aligned}$$

In this case we will denote $E^1$ by $E^s$ and call it the contracting direction. Also $E^2$ will be denoted by $E^{cu}$ and called the center-unstable direction.

#### Definition 3.3

Let *G* be a $C ^{\infty }$ vector field on $\mathbb {R}^3$ with a Lorenz-like equilibrium; that is, the eigenvalues of $DG_p$ are real and satisfy $0<-\lambda _s<\lambda _u<-\lambda _{ss}$. We say that *G* is **strongly dissipative** if the divergence of the vector field *G* is strictly negative; that is, there is $c>0$ such that ${\text {Div}}(G)(x)\le -c$ for every *x* and the eigenvalues of the singularity at *p* satisfy the constraint

21 $$\begin{aligned} \lambda _u+\lambda _{ss}<\lambda _s. \end{aligned}$$

Figure 2 shows how the flows given by the Neutral model 1 and 2 send the lines in the *y*-direction in $\Sigma$ to lines in the *y*-direction in *S*. Thus, its derivative $D{\text {N}}^t$ preserves the lines in the *y*-direction. Furthermore, by the way the flow from *S* to $\Sigma$ was constructed (Fig. 1) we notice that horizontal lines in *S*; that is, parallel to the *y*-axis, are taken to parallel lines to the same axis in $\Sigma$. In other words, the flow from *S* to $\Sigma$ preserves parallel lines to the *y*-axis. Since this flow is a composition of a rotation, an expansion and a translation, the derivative of the flow also preserves planes orthogonal to the *y*-axis. From this we can deduce that the splitting $\mathbb {R}^3=E\oplus F$, where $E=\{0\}\times \mathbb {R}\times \{0\}$ and $F=\mathbb {R}\times \{0\}\times \mathbb {R}$, is preserved by the flow; i.e., $D{\text {N}}^t(E)=E$ and $D{\text {N}}^t(F)=F$ for any *t*, where ${\text {N}}^t$ is the flow obtained from Equation (4) with the corresponding parameters for model 1 and 2. Since we have uniform contraction along *E* ($\left\Vert D{\text {N}}^t|_{E_x}\right\Vert _{}\le {\text {e}}^{\lambda _{ss}t}$ with $\lambda _{ss}=-\ell <0$ for every $x\in U$) and domination of the splitting ($\left\Vert D{\text {N}}^t|_{E_x}\right\Vert _{}\cdot \left\Vert D{\text {N}}^{-t}|_{F_{{\text {N}}^t(x)}}\right\Vert _{}\le {\text {e}}^{\lambda _{ss}-\lambda _s t}$ with $\lambda _{ss}-\lambda _s<0$ for every $x\in U$) we can conclude that *U* is partially hyperbolic. It is worth noticing that the origin is the only point that spoils the singular hyperbolicity condition (a set *A* is singular hyperbolic if all its singularities are hyperbolic and it has volume expanding central direction) since $J^{cu}_t({\bar{0}})=|\det D{\text {N}}^t|_{F_0}|={\text {e}}^{(\lambda _u+\lambda _s)t}=1$ (recall $\lambda _s=0=\lambda _u$); that is, there is no area expansion along the subbundle *F*. Hence, $\Lambda _{{\text {Neu}}}$ is a partially hyperbolic attractor.

Theorem 6 in [6] provides us with local strong-stable and center-unstable laminations $$W^{ss}_\epsilon (x)$$ and $$W^{cu}_\epsilon (x)$$, respectively, through the points $$x\in \Lambda _{{\text {Neu}}}\setminus \{{\bar{0}}\}$$. We note that both, $$W^{ss}_\epsilon (x)$$ and $$W^{cu}_\epsilon (x)$$, are embedded discs and hence sub-manifolds of *M*. Also $$W^{ss}_\epsilon (x)$$ is uniquely determined since $$E^s$$ is uniformly contracting. Corollary 6 in [6] shows us that the local strong-stable lamination can be extended to an invariant foliation $$\mathcal {F}^{ss}(x)$$ of a open neighbourhood of $$\Lambda _{{\text {Neu}}}$$ with $$ C ^{2}$$ leaves and whose foliated charts are $$ C ^{1}$$. Moreover, the leaves are uniformly contracted by the action of the flow.

We note that $$\Sigma $$ is a $$ C ^{2}$$ embedded compact disk transversal to the flow $${\text {N}}^t$$. Furthermore, $$\Sigma $$ is contained in the open neighbourhood *V* of $$\Lambda _{{\text {Neu}}}$$. By Theorem 6 and Corollary 6 from [6] we know that local strong-stable lamination $$W^{ss}_\epsilon (x)$$ extends to an invariant foliation $$\mathcal {F}^{ss}(x)$$. In this way, for $$x\in \Sigma $$ we define $$W^{ss}(x,\Sigma )$$ to be the connected component of $$\mathcal {F}^{sc}(x)\cap \Sigma $$, where $$\mathcal {F}^{sc}(x)=\bigcup _{t\in \mathbb {R}}{\text {N}}^t(\mathcal {F}^{ss}(x))$$ is the center-stable leaf. Since the flow $$({\text {N}}^t)_{t\in \mathbb {R}}$$ is $$ C ^{2}$$, $$W^{ss}(x,\Sigma )$$ is a $$ C ^{2}$$ one-dimensional embedded curve for every $$x\in \Sigma $$ and their leaves form a $$ C ^{1}$$ foliation $$\mathcal {F}^{ss}_\Sigma $$ of $$\Sigma $$.

**Fig. 5:**
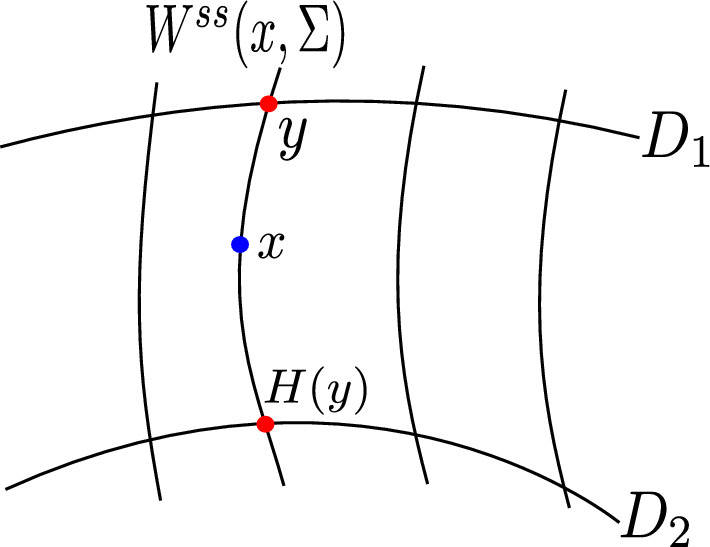
The holonomy map

Given a pair of embedded disks $D_1$ and $D_2$ in $\Sigma$ intersecting transversally a set $\{W^{ss}(x,\Sigma )\}_{x\in \Sigma }$ of stable leafs, the **holonomy map** $H : D_1\cap W^{ss}(x,\Sigma ) \rightarrow D_2\cap W^{ss}(x,\Sigma )$ assigns to $y\in D_1\cap W^{ss}(x,\Sigma )$ the unique point in $h(y)\in D_2\cap W^{ss}(x,\Sigma )$, see Fig. 5.

From the developments in the partially hyperbolicity theory by Brin-Pesin [13] and Pugh-Shub [32] we have that the projection along leaves, also known as holonomies, between pair of transversal surfaces to $\mathcal {F}^{ss}$ have a Hölder continuous Jacobian with respect to Lebesgue measure. This implies a similar conclusion for the holonomies transversal to $\mathcal {F}^{cs}$. It follows that the holonomy between pairs of transversal curves to $\mathcal {F}_\Sigma ^{ss}$ along the lines of $\mathcal {F}^{ss}_\Sigma$ can be thought of as interval maps having a Hölder Jacobian. Hence these holonomies are $C ^{1+\alpha }$ for some $0<\alpha <1$. In this setting, the leaves $W^ss(x,\Sigma )$, with $x\in \Sigma$, determine a foliation $\mathcal {F}^{ss}_\Sigma$ of $\Sigma$ with transversal smoothness $C ^{1+\alpha }$.

Therefore, we can assume that $$\Sigma $$ is the image of the unit square $$I\times I$$ under the action of a $$ C ^{1+\alpha }$$ diffeomorphism *h*, for some $$0<\alpha <1$$. Furthermore, *h* sends vertical lines inside the leaves of $$\mathcal {F}^{ss}_\Sigma $$. The next step is to prove that the strong stable foliation $$\mathcal {F}^{ss}(x)$$ is not only $$ C ^{1}$$ but also $$ C ^{1+\alpha }$$, for some $$0<\alpha <1$$. This was done by Araújo, Melbourne and Varandas in [7], stated as Lemma 2.2. This result is a consequence of domination of the splitting (Equation (19)), uniform contraction along the stable direction (Equation (20)) and strong dissipativity (Definition 3.3). Therefore, we can conclude that the neutral geometrical flow has a strong-stable foliation $$\mathcal {F}^{ss}(x)$$ which is $$ C ^{1+\alpha }$$. Furthermore, the modified return map also has a strong-stable foliation $$\mathcal {F}^{ss}_\Sigma $$, whose transversal smoothness is $$ C ^{1+\alpha }$$, with $$0<\alpha <1$$.

A final remark concerning this subsection, Theorem 6 in [6] is stated for a singular hyperbolic attractor. However, the conclusions and arguments still hold true if we consider a compact partially hyperbolic invariant set instead of a singular hyperbolic set. We will also like to mention that the situation for the origin is slightly different, since the splitting of the tangent bundle is given by a one-dimensional strong-stable direction $E^s$ and a two-dimensional center direction $E^c$. However, we are only concerned with the existence of the strong-stable manifold $W^{ss}({\bar{0}})$, which follows from the theory of partial hyperbolicity.

### The UNI Condition

The main goal of this subsection is to show that the stable and unstable manifolds of the modified geometrical model are jointly nonintegrable. The joint nonintegrability of stable and unstable foliations can be interpreted as follows: The stable and unstable foliation of an Anosov flow are always transversal, therefore, if they are jointly integrable, this provides us with a codimension one invariant foliation which is transversal to the flow direction. In contrast, if there exists a codimension one submanifold transversal to the flow direction, then this foliation must be subfoliated by both the stable and unstable foliations. Thus they must be jointly integrable. In this situation it is known [23, Proposition 3.3] that the flow is semiconjugate to a suspension with a locally constant roof-function over a subshift of finite type. Such a flow need not mix! From the work of Araújo, Butterley and Varandas [8], we know that the joint nonintegrability of the stable and unstable manifolds is equivalent to the uniformly nonintegrability (UNI) condition. As we will see later in this section, the UNI condition, stated formally in Definition 3.7, will ensure that the roof function of the suspension flow, which we will use to model the Neutral geometrical Lorenz flow, is not cohomologous to a constant function; that is, the time it takes for a point in the base to reach the roof of the suspension flow is not the same for every point. This property will guarantee the mixing properties.

In this chapter we will aim to prove the UNI condition. In [5] and [7] Araújo *et al. * prove the UNI condition by exploiting the properties obtained by using hyperbolic times. From now on, when we talk about the neutral geometrical model, we will refer to both models we constructed in Sect. 2.

Let $$I=[-1,1]$$ and $$f_{{\text {Neu}}} : I \rightarrow I$$ be the one-dimensional Lorenz-like map obtained from the neutral geometrical model. We notice that $$\{0\}$$ is a nondegenerate^2^ exceptional set for $$f_{{\text {Neu}}}$$. From Theorem 4.3 in [5] we know that there are $${\tilde{X}}$$ a neighbourhood of the singularity 0, a countable partition $$\tilde{\mathcal {Q}}$$ of $${\tilde{X}}$$ Lebesgue modulo zero into subintervals, a function $$\tau : {\tilde{X}} \rightarrow \mathbb {Z}^+$$ constant on partition elements and the induced map $${\tilde{F}}=f_{{\text {Neu}}}^\tau : {\tilde{Q}} \rightarrow {\tilde{X}}$$ which is a $$ C ^{2}$$ uniformly expanding diffeomorphism with bounded distortion for any $${\tilde{Q}}\in \tilde{\mathcal {Q}}$$.

The motivation behind taking the inducing scheme is that we aim to extend the Gibbs-Markov map ${\tilde{F}}$ to a two-dimensional Gibbs- Markov map *F* and build the suspension flow $F^t$ over the map *F* with roof function *R* (see Equation (22)). This will allow us to use the results given in [12] to deduce the decay of correlations for the suspension flow $F^t$ and ultimately for the geometrical neutral Lorenz flow ${\text {N}}^t$. Furthermore, we want to use the properties provided by the hyperbolic times and the bounded distortion of the map ${\tilde{F}}$, to obtain the bound stated in Proposition 3.9 for the roof function *R*, which will help us prove the UNI condition for the geometrical neutral flow. We make now some observations regarding the induced map ${\tilde{F}}$.

#### Observation 3.4

The map ${\tilde{F}}$ is obtained by inducing $f_{{\text {Neu}}}$ on the interval ${\tilde{X}}$, and the inducing time is given by the sum of a hyperbolic time with a non-negative integer bounded by *N*, where *N* is such that $\bigcup _{i=1}^N(f_{{\text {Neu}}}^i)^{-1}(\{0\})$ is $2\delta$-dense in ${\tilde{X}}$. Furthermore, ${\tilde{F}}$ is a full branch Markov map onto ${\tilde{X}}$ since 0 has dense preimages under $f_{{\text {Neu}}}$. For more details we refer the reader to [5].

In addition to the bounded distortion and uniform expansion of ${\tilde{F}}$, we have the following inequalities for $f_{{\text {Neu}}}$ as a consequence of hyperbolic times. Given $\sigma \in (0,1)$ and $c>0$, there is a constant $b>0$ such that:

1. (Backward contraction) Let ${\tilde{Q}}(x)$ denotes the element of the partition $\tilde{\mathcal {Q}}$ containing *x*, for $y\in {\tilde{Q}}(x)$
  $$\begin{aligned} |f_{{\text {Neu}}}^i(y)-f_{{\text {Neu}}}^i(x)|\le b\sigma ^{\tau (x)-i}|{\tilde{F}}(y)-{\tilde{F}}(x)|, \;\text { }\; i=0,\ldots ,\tau (x)-1. \end{aligned}$$
2. (Slow recurrence to the singular point)
  $$\begin{aligned} |f_{{\text {Neu}}}^i(x)|\ge \sigma ^{c(\tau (x)-i)}, \;\text { }\; i=0,\ldots ,\tau (y)-1. \end{aligned}$$

Following [7], our next step is to construct a piecewise uniformly hyperbolic map *F* with infinitely many branches, which covers ${\tilde{F}}$. First, let $W^{ss}_{P_{{\text {Neu}}}}(x)$ denote the stable leaf under the Poincaré map $P_{{\text {Neu}}}$ containing the point *x*, $\pi : \Sigma \rightarrow I$ the projection map. We define $X=\bigcup \{W^{ss}_{P_{{\text {Neu}}}}(x)\;|\;x\in {\tilde{X}}\}$ as the union of stable leaves along ${\tilde{X}}$. We also extend the induced time $\tau$ to a function on *X* denoted also by $\tau$ and given by $\tau (x)=\tau (\pi (x))$. We make the following important observation on the tails of $\tau$.

#### Observation 3.5

The tails of the return time $\tau$ and its extension also denoted by $\tau$ are exponential (see [33]); i.e., there exists a constant $c>0$ such that $\mu _{{\tilde{X}}}(\tau >n)=\mathcal {O}({\text {e}}^{-cn})$ and $\mu _{X}(\tau >n)=\mathcal {O}({\text {e}}^{-cn})$, where $\mu _{{\tilde{X}}}$ and $\mu _{X}$ are the SRB-measures of the Gibbs-Markov maps *F* and ${\tilde{F}}$, see below.

Now, we construct $F : X \rightarrow X$ the Poincaré map by setting $F(x)=P_{{\text {Neu}}}^{\tau (\pi (x))}(x)$ for $x\in X$. Furthermore, let $\mathcal {Q}$ be the measurable partition of *X* by taking $\bigcup \{W^{ss}_{P_{{\text {Neu}}}}(x)\;|\;x\in {\tilde{Q}}\}$ as its elements, with ${\tilde{Q}}\in \tilde{\mathcal {Q}}$. We will make use of *X* and *F* when making the model of the neutral geometrical Lorenz flow by a suspension flow.

It is standard [33] that the map ${\tilde{F}}$ has a unique a.c.i.p measure $\mu _{{\tilde{X}}}$ on ${\tilde{X}}$. Furthermore, $r_{{\text {Neu}}}\in {\text {L}}^{1}(\mu _{{\tilde{X}}})$ and there exists a unique invariant measure $\mu _X$ for *F*, $\mu _{\Sigma }$ for $P_{{\text {Neu}}}$ and $\mu _{I}$ for $f_{{\text {Neu}}}$ satisfying $\pi _*(\mu _X)=\mu _{{\tilde{X}}}$, $\pi _*(\mu _\Sigma )=\mu _{I}$, and also

$$\begin{aligned} \mu _\Sigma= & {} \sum _{n\ge 1}\sum _{i=0}^{n-1}(P_{{\text {Neu}}}^i)_*(\mu _X|\{\tau \circ \pi =n\}), \\ \mu _I= & {} \sum _{n\ge 1}\sum _{i=0}^{n-1}(f_{{\text {Neu}}}^i)_*(\mu _{{\tilde{X}}}|\{\tau \circ \pi =n\}). \end{aligned}$$

Moreover, $\mu _X\ll \mu _\Sigma$ and $\mu _X(X)=1$, thus $\mu _\Sigma (X)>0$. We take the induced roof function $R : X \rightarrow \mathbb {R}^+$ given by

22 $$\begin{aligned} R(x)=\sum _{k=0}^{\tau (x)-1}r_{{\text {Neu}}}(P_{{\text {Neu}}}^k(x)). \end{aligned}$$

Notice that *R* is constant along stable leaves because $r_{{\text {neu}}}$ is constants along stable leaves. We also call *R* the quotient induced roof function $R : {\tilde{X}} \rightarrow \mathbb {R}^+$. With this in mind we can state the definition of the UNI condition. First, we give the definition of suspension flow.

#### Definition 3.6

Let $(\Sigma ,\nu )$ be a probability space and $P : \Sigma \rightarrow \Sigma$ an ergodic measure-preserving transformation. Let $r : \Sigma \rightarrow \mathbb {R}^+$ be a measurable (Hölder continuous) roof function. We define the **suspension space** as $\Sigma ^r=\{(x,u)\in \Sigma \times [0,r(x)]\}/\sim$, where $(x,r(x))\sim (P(x),0)$. The **suspension flow** $f_t : \Sigma ^r \rightarrow \Sigma ^r$ is given by $f_t(x,u)=(x,u+t)$ computed modulo identifications and the measure $\mu =\nu \times \lambda$, where $\lambda$ is the Lebesgue measure, is ergodic and $f_t$-invariant. In the finite measure case, we normalise by ${\bar{r}}=\int _\Sigma rd\mu$ so that $\mu =\frac{\nu \times \lambda }{{\bar{r}}}$ is a probability measure.

#### Definition 3.7

Let $R : X \rightarrow \mathbb {R}^+$ be a roof function as above, $F^t : X^R \rightarrow X^R$ the suspension flow built over $F : X \rightarrow X$, $R_n(x)=\sum _{i=0}^{n-1}R\circ F^i(x)$. Define $\psi _{h_1,h_2}=R_n\circ h_1-R_n\circ h_2 : X \rightarrow \mathbb {R}$, for $h_1,\;h_2\in \mathcal {H}_n$; that is, inverse branches of $F^n$. Then the **UNI condition** holds if there exist $D>0$ and $h_1,\;h_2\in \mathcal {H}_{n_0}$ for some sufficiently large $n_0\ge 1$, such that $|\psi '_{h_1,h_2}|\ge D$.

**Fig. 6:**
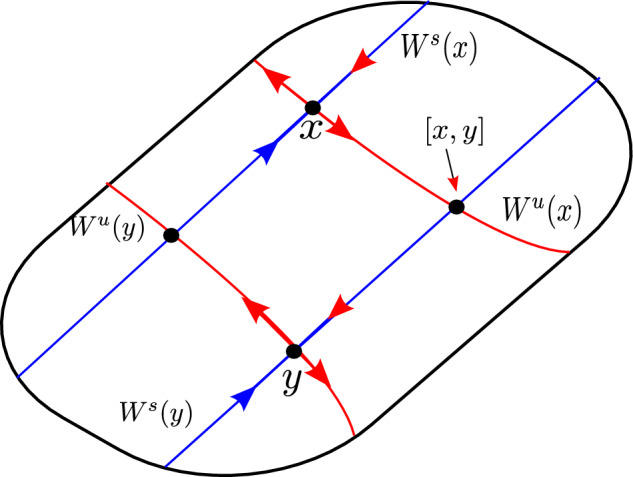
Local product structure for *F*

We saw in the previous subsection that the Poincaré map $P_{{\text {Neu}}} : \Sigma \rightarrow \Sigma$ has a strong stable foliation. We observe that the leaves of this foliation cross $\Sigma$, hence the induced map *F* has a strong stable manifold $W^{ss}_F(x)=W^{ss}_{P_{{\text {Neu}}}}(x)$ that crosses *X*. Araújo et al. in [7, Proposition 2.4] provide us with local unstable manifolds of uniform size for *F* and defined almost everywhere, and by [7, Proposition 2.4] we obtain a local product structure, see Fig. 6.

To use the arguments given in [7] for the temporal distortion function we need to adapt the proofs of the uniform bound of the derivative of the induced roof function *R* and a bound on the flow time $r_{{\text {Neu}}}$. An adjustment is required because we are changing the flow time from $\Sigma$ to *S*. Recall that the original flow time for the geometrical Lorenz model is given by (3), whereas the modified flow time is given by (6); that is, we change from logarithmic to polynomial. More precisely, we have the following propositions.

#### Proposition 3.8

Let ${\tilde{Q}}\in \tilde{\mathcal {Q}}$ and ${\tilde{F}} : {\tilde{Q}} \rightarrow {\tilde{X}}$ as above. Denote by $\mathcal {H}$ the set of all inverse branches of ${\tilde{F}}$. Then we have

$$\begin{aligned} \sup _{h\in \mathcal {H}}\sup _{x\in {\tilde{X}}}|D(R\circ h)(x)|<\infty . \end{aligned}$$

#### Proof

Let ${\tilde{Q}}\in \tilde{\mathcal {Q}}$ and $h\in \mathcal {H}$; that is, $h : {\tilde{X}} \rightarrow {\tilde{Q}}$ be an inverse branch of ${\tilde{F}}$ with inducing time $\tau =\tau ({\tilde{Q}})\ge 1$ and fix $x\in {\tilde{Q}}$. We first observe that

$$\begin{aligned} |D(R\circ h)(x)|= & {} |DR(h(x))|\cdot |Dh(x)|=\frac{|DR(h(x))|}{|D{\tilde{F}}(h(x))|}\\= & {} \left| \sum _{i=0}^{\tau -1}\frac{(Dr_{{\text {Neu}}}\circ f_{{\text {Neu}}}^i)\cdot Df_{{\text {Neu}}}^i}{D{\tilde{F}}}\circ h(x)\right| . \end{aligned}$$

From the construction of the inducing partition using hyperbolic times (for more details on hyperbolic times see [2] and [5]), we have that backward contraction and slow recurrence to the singular point, see Observation 3.4; that is, there are constants $\sigma \in (0,1)$, $b_0>0$ and $c \in (0,\frac{1}{2}]$ such that

1. (Backward contraction) For $y\in {\tilde{Q}}(x)$
  $$\begin{aligned} |f_{{\text {Neu}}}^i(y)-f_{{\text {Neu}}}^i(x)|\le b_0\sigma ^{\tau -i}|{\tilde{F}}(y)-{\tilde{F}}(x)|, \;\text { }\; i=0,\ldots ,\tau -1. \end{aligned}$$
2. (Slow recurrence to the singular point)
  $$\begin{aligned} |f_{{\text {Neu}}}^i(x)|\ge \sigma ^{c(\tau -i)}, \;\text { }\; i=0,\ldots ,\tau -1. \end{aligned}$$

Notice that

23 $$\begin{aligned} \left| \frac{Df_{{\text {Neu}}}^i}{D{\tilde{F}}}\circ h(x)\right| \le b_0\sigma ^{\tau -i}\;\text { }\; i=0,\ldots ,\tau -1, \end{aligned}$$

where the inequality follows from the backward contraction.

Moreover, from the slow recurrence to the singularity we also get the following inequality;

24 $$\begin{aligned} |(Dr_{{\text {Neu}}}\circ f_{{\text {Neu}}}^i)\circ h(x)|\le b_1\sigma ^{-c(1+\frac{1}{\beta _2})(\tau -i)}\;\text { }\; i=0,\ldots ,\tau -1, \end{aligned}$$

for some constant $b_1>0$. Altogether, Equations (23) and (24) imply that

25 $$\begin{aligned} |D(R\circ h)(x)|\le b\sum _{i=0}^{\tau -1}\sigma ^{si}, \end{aligned}$$

where $s=1-c(1+\frac{1}{\beta _2})$. Since $0<c\le \frac{1}{2}$ and $\beta _2>2$, we have $s<1$. Therefore the sum converges, and we have that

$$\begin{aligned} \sup _{h\in \mathcal {H}}\sup _{x\in {\tilde{X}}}|D(R\circ h)(x)|<\infty . \end{aligned}$$

$$\square $$

For $x,\;y\in {\tilde{X}}$ we define the separation time *s*(*x*, *y*) as the least integer $n\ge 0$ such that ${\tilde{F}}^n(x)$ and ${\tilde{F}}^n(y)$ are in different partition elements of $\mathcal {Q}_0$. For given $0<\eta <1$, the symbolic metric is defined on ${\tilde{X}}$ as $d_\eta (x,y)=\eta ^{s(x,y)}$. Finally, we set $r_{{\text {Neu}}}^{(k)}(x)=\sum _{i=0}^{k-1}r_{{\text {Neu}}}(P_{{\text {Neu}}}^i(x))$.

#### Proposition 3.9

There exists $B>0$ such that for all $x,\; y\in {\tilde{X}}$ with $s(x,y)\ge 1$ and $0\le k\le \tau (x)=\tau (y)$ we have $|r^{(k)}_{{\text {Neu}}}(x)-r^{(k)}_{{\text {Neu}}}(y)|\le B|{\tilde{F}}(x)-{\tilde{F}}(y)|^\epsilon$. Consequently, there is $\eta \in (0,1)$ such that $|R|_\eta <\infty$, where $|R|_\eta =\sup _{x\ne y}\frac{|R(x)-R(y)|}{d_\eta (x,y)}$ denotes the Lipschitz constant of the quotient induced roof function $R : {\tilde{X}} \rightarrow \mathbb {R}^+$ with respect to $d_\eta$. Moreover, $|{\tilde{F}}(x)-{\tilde{F}}(y)|\le Bd_\eta (x,y)$.

#### Proof

For convenience, in this proof *f* will denote $f_{{\text {Neu}}}$. Let $x,\;y\in {\tilde{X}}$ such that $s(x,y)=n\ge 1$ and $0\le k\le \tau (x)=\tau (y)$. Thus $y\in {\tilde{Q}}^n(x)$, where ${\tilde{Q}}^n(x)=\displaystyle \bigvee _{i=0}^{n-1}({\tilde{F}}^i)^{-1}({\tilde{Q}}(x))$ is the *n*th refinement of ${\tilde{Q}}(x)$, and so $\tau ({\tilde{F}}^i(x))=\tau ({\tilde{F}}^i(y))$ for $i=0,\ldots ,n-1$. Hence, the choice of the cross- section assures that $r_{{\text {Neu}}}$ is constant along stable leaves and that $r_{{\text {Neu}}}(x)=|x|^{-\frac{1}{\beta _2}}h(x)+\tau _2(x)$, where *h*(*x*) is bounded and bounded away from zero. In fact, *h*(*x*) is of the form $h_0 + \mathcal {O}(|x|^\gamma )$ where $h_0$ is a positive constant and $\gamma > 0$ depending on whether the higher order terms are consider in (7) or not. Also *h*(*x*) is differentiable for $x > 0$, because the Dulac map is differentiable. Then we can write

$$\begin{aligned} |R(x)-R(y)|\le & {} \sum _{i=0}^{\tau (x)-1}|r_{{\text {Neu}}}(f^i(x))-r_{{\text {Neu}}}(f^i(y))|\\\le & {} \sum _{i=0}^{\tau (x)-1}\left| |f^i(x)|^{-\frac{1}{\beta _2}}h(f^i(x))-|f^i(y)|^{-\frac{1}{\beta _2}}h(f^i(y))\right| \\{} & {} +|\tau _2(f^i(x)-\tau _2(f^i(y))|. \end{aligned}$$

We first notice that,

26 $$\begin{aligned} \left| \tau _2(f^i(y))-\tau _2(f^i(x))\right|\le & {} \left\Vert \tau _2\right\Vert _{\epsilon }\left| f^i(y)-f^i(x)\right| ^\epsilon \nonumber \\\le & {} \sigma ^{\epsilon (\tau (x)-i)}\left\Vert \tau _2\right\Vert _{\epsilon }\left| {\tilde{F}}^i(y)-{\tilde{F}}^i(x)\right| ^\epsilon . \end{aligned}$$

The second inequality in (26) follows from Observation 3.4-1. Now, denote $\Bigg | |f^i(x)|^{-\frac{1}{\beta _2}}h(f^i(x)) - |f^i(y)|^{-\frac{1}{\beta _2}}h(f^i(y))\Bigg |$ by *A*, then we obtain that,

27 $$\begin{aligned} A\le & {} \left| |f^i(x)|^{-\frac{1}{\beta _2}} - |f^i(y)|^{-\frac{1}{\beta _2}} \right| |h(f^i(x))| \nonumber \\{} & {} + |f^i(y)|^{-\frac{1}{\beta _2}} |h(f^i(x))-h(f^i(y))|. \end{aligned}$$

We notice that $\left| |f^i(x)|^{-\frac{1}{\beta _2}} - |f^i(y)|^{-\frac{1}{\beta _2}}\right|$ is bounded. Indeed we have the following:

28 $$\begin{aligned} \left| |f^i(x)|^{-\frac{1}{\beta _2}} - |f^i(y)|^{-\frac{1}{\beta _2}}\right|= & {} |f^i(x)|^{-\frac{1}{\beta _2}} \left| 1 - \left( \frac{ |f^i(y)|}{|f^i(x)|} \right) ^{-\frac{1}{\beta _2}}\right| \nonumber \\\le & {} |f^i(x)|^{-\frac{1}{\beta _2}} \left| 1 - \left( 1 + \frac{ |f^i(y)-f^i(x)|}{|f^i(x)|} \right) ^{-\frac{1}{\beta _2}}\right| \nonumber \\\le & {} |f^i(x)|^{-\frac{1}{\beta _2}} \frac{C_0}{\beta _2} \frac{ |f^i(y)-f^i(x)|}{|f^i(x)|} \nonumber \\= & {} \frac{C_0}{\beta _2} |f^i(x)|^{-(1+\frac{1}{\beta _2})} |f^i(y)-f^i(x)| \nonumber \\\le & {} \frac{C_0}{\beta _2} \sigma ^{\left( 1-c\left( 1+\frac{1}{\beta _2}\right) \right) (\tau (x)-i)} \left| {\tilde{F}}(y)-{\tilde{F}}(x)\right| , \end{aligned}$$

where $C_0>0$ and $0<c\le \frac{2}{3}$. The first and second inequalities follow from the Bernoulli inequality and from Observation 3.4-1 and 2, respectively. Since $h(f^i(x))$ is bounded and by (28), we can bound the first term in the sum of (27) by $\frac{C_0}{\beta _2} \sigma ^{(1-c(1+\frac{1}{\beta _2}))(\tau (x)-i)}\left| {\tilde{F}}(y)- {\tilde{F}}(x)\right|$.

To finish the proof it remains to find a bound for

29 $$\begin{aligned} |f^i(y)|^{-\frac{1}{\beta _2}} |h(f^i(x))-h(f^i(y))|. \end{aligned}$$

Notice that $|h(f^i(x))-h(f^i(y))|\approx h'(\xi )|f^i(x)-f^i(y)|$, with $f^i(x)<\xi <f^i(y)$. Hence, by Observations 3.4-1 and 2, we have that (29) is bounded by $C_0h'(\xi )\sigma ^{(1-\frac{c}{\beta _2})(\tau (x)-i)}|{\tilde{F}}(y)-{\tilde{F}}(x)|$. Assuming that $h'(\xi )$ is bounded and combining all the previous bounds we have that,

$$\begin{aligned} |R(x)-R(y)|\le & {} C\sum _{i=0}^{\tau (x)-1}[ (\sigma ^{s(\tau (x)-i)}+\sigma ^{u(\tau (x)-i)})|{\tilde{F}}(y)-{\tilde{F}}(x)|\\{} & {} +\ \sigma ^{\epsilon (\tau (x)-i)}\left\Vert \tau _2\right\Vert _{\epsilon }|{\tilde{F}}^i(y)-{\tilde{F}}^i(x)|^\epsilon ]\\\le & {} B |{\tilde{F}}^i(y)-{\tilde{F}}^i(x)|^\epsilon , \end{aligned}$$

for some constant $B>0$ where $s=1-c(1+\frac{1}{\beta _2}) > 0$ and $u=1-\frac{c}{\beta _2}$. As in the previous proof, the sum converges since $\beta _2>2$, $0<c\le \frac{2}{3}$ and hence $0<s,u<1$. This establishes what we were aiming to prove.

One caveat we have to make here is that this argument is only valid if we assume boundedness for $h'$. This is true if the higher order terms in (7) are not present. If higher order terms are present, boundedness of $h'$ is plausible, but since the required perturbation argument in [17] is less constructive so as to immediately derive this boundedness, we will try to convince the reader with the numeric analysis performed at the end of this work that this boundedness is indeed true. Thus, rather than a rigorous proof we give a combination of mathematical arguments and numerical verification. $\square$

**Fig. 7:**
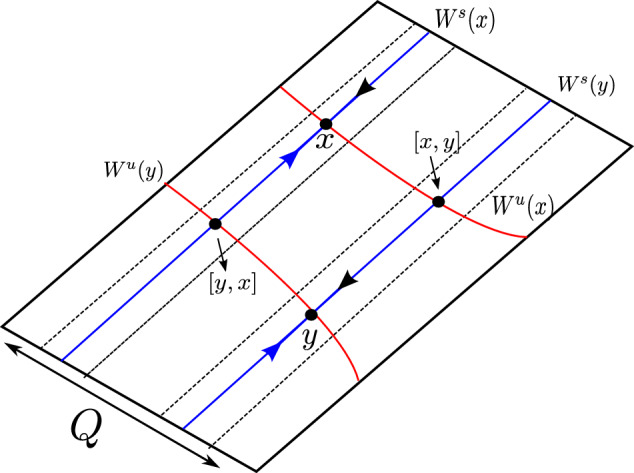
Local product structure for *F*

Now let $Q\in \mathcal {Q}$ be a partition elements for *F*. The temporal distortion function $T : Q\times Q \rightarrow \mathbb {R}$ is defined almost everywhere by,

30 $$\begin{aligned} T(x,y)= & {} \sum _{i=-\infty }^\infty [r_{{\text {Neu}}}(P_{{\text {Neu}}}^i(x))-r_{{\text {Neu}}}(P_{{\text {Neu}}}^i([x,y]))-r_{{\text {Neu}}}(P_{{\text {Neu}}}^i([y,x]))\nonumber \\{} & {} +\ r_{{\text {Neu}}}(P_{{\text {Neu}}}^i)(y)]\nonumber \\= & {} \sum _{i=-\infty }^{-1}[r_{{\text {Neu}}}(P_{{\text {Neu}}}^i(x))-r_{{\text {Neu}}}(P_{{\text {Neu}}}^i([x,y]))-r_{{\text {Neu}}}(P_{{\text {Neu}}}^i([y,x]))\nonumber \\{} & {} +\ r_{{\text {Neu}}}(P_{{\text {Neu}}}^i(y))], \end{aligned}$$

where [*x*, *y*] is the local product of *x* and *y* (see Fig. 7). The second inequality follows from the property of $r_{{\text {Neu}}}$ of being constant along stable leaves.

Now, for every $x,\; y\in X$ in the same unstable manifold for $F : X \rightarrow X$ we define

31 $$\begin{aligned} T_0(x,y)=\sum _{i=1}^\infty [r_{{\text {Neu}}}(P_{{\text {Neu}}}^{-i}(x))- r_{{\text {Neu}}}(P_{{\text {Neu}}}^{-i}(y))]. \end{aligned}$$

The continuity and other properties of $T_0$ are stated in [7, Lemma 3.1]. Furthermore, we can rewrite the temporal distortion function *T*(*x*, *y*) in terms of $T_0$; that is,

$$\begin{aligned} T(x,y)=T_0(x,[x,y])+T_0(y,[y,x]). \end{aligned}$$

The main result concerning the temporal distortion function establishes the joint nonintegrability of the stable and unstable foliations for the flow by proving that the temporal distortion function *T* is not identically zero, that is, there is $Q\in \mathcal {Q}$ and $x,\; y\in Q$ such that $T(x,y)\ne 0$ and is stated and proven in [7, Theorem 3.4].

We adjusted the proof of the uniform bound of the derivative of the induced roof function for the geometrical neutral Lorenz model since we want to apply [9, Proposition 7.4] in order to use the same arguments given in [3, Corollary 4.3]. Thus, we get the UNI condition for the geometrical neutral Lorenz flow. For completeness we state it in the following theorem.

#### Theorem 3.10

The UNI condition holds for the geometrical neutral Lorenz flow.

For fixed $x\in X$, we define the map $h : W^{u}_F(x) \rightarrow \mathbb {R}$ given by

$$\begin{aligned} h(y)=T(x,y)=T_0(x,[x,y])+T_0(y,[y,x]), \end{aligned}$$

the map *h* is $$ C ^{1}$$. Furthermore, there exists a nonempty open set $$U\subset W^u_F(x)$$ such that $$h|_U$$ is a $$ C ^{1}$$ diffeomorphism. For the proofs of the properties of the map *h* see Proposition 3.6 and Corollary 4.7 in [7].

The next result will be of great help in proving the decay of correlations for the geometrical neutral Lorenz flow. The original statement involves the geometrical Lorenz flow, but the same arguments can be used to prove the same result for our setting. Before stating the result we give a definition.

#### Definition 3.11

Let *X* and *F* be as in the beginning of Section 6.3. A subset $Z_0\subset X$ is called a **finite subsystem**of *X* if $Z_0=\bigcap _{n\ge 0}F^{-n}Z$, where *Z* is the union of finitely many partition elements of *X*.

Let $Q_1$ and $Q_2\in \mathcal {Q}$ be two partition elements and consider $Q=Q_1\cup Q_2$. We define the finite subsystem $Q_0=\bigcap _{n\ge 0}F^{-n}Q$, then we have the following:

#### Proposition 3.12

[7, Proposition 3.8]   For the finite subsystem $Q_0$, the set $T(Q_0\times Q_0)$ has positive lower box dimension.

We will like to end this Section with the following remark. To establish their results on decay of correlations, Bálint et al. in [12] and Melbourne in [30] assumed a very important, yet technical property namely, absence of approximate eigenfunctions. They also provide some criteria that guarantees the absence of approximate eigenfunctions. The first one, involves the temporal distortion function providing a nonintegrability condition. This criteria is given by Proposition 3.12; that is, when the temporal distortion function is not identically zero. In other words, when the UNI condition is satisfied. The second one, is a Diophantine condition on the periods of three periodic solutions [21], which is satisfied with probability one. It is important to remark that from these criteria, the UNI condition is robust while the Diophantine condition is not.

## Decay of Correlations

In this section we prove Theorem 1.1 for the first two neutral models. The third and more general model will be analysed in Sect. 5. We will use the results of Bálint *et al. * [12] to prove our theorem. In [12] polynomial decay of correlations for non-uniformly hyperbolic flows is proven under absence of approximate eigenfunctions. Let us start by giving the description of a non-uniformly hyperbolic flow described in [12].

First, we observe that the geometrical neutral Lorenz flow ${\text {N}}^t : \Lambda _{{\text {Neu}}} \rightarrow \Lambda _{{\text {Neu}}}$, where $\Lambda _{{\text {Neu}}}$ is the geometrical neutral Lorenz attractor, can be modelled as the suspension flow $S^t : \Sigma ^{r_{{\text {Neu}}}} \rightarrow \Sigma ^{r_{{\text {Neu}}}}$ over the Poincaré map $P_{{\text {Neu}}}$ with base the cross-section $\Sigma$ and roof function $r_{{\text {Neu}}}$ from (6). However, in order to use the results of [12], we take the alternative model $F^t : X^R \rightarrow X^R$, where $X\subset \Sigma$ is a cross-section to the flow with nice hyperbolic structure (local product structure) and with induced roof function $R : X \rightarrow \mathbb {R}^+$ given by $R(x)=\sum _{k=0}^{\tau (x)-1}r_{{\text {Neu}}}(P_{{\text {Neu}}}^k(x))$, see Sect. 3.2. Then the suspension flow $F^t$ built over the uniformly hyperbolic map $F : X \rightarrow X$ is identical to the suspension flow $S_t$, thus $F_t$ is an extension of the underlying flow, namely the neutral geometrical Lorenz flow. Within this framework, ${\text {N}}^t$ is called in [12] a **non-uniformly hyperbolic flow**.

Under suitable conditions it can be shown that the suspension flow $F^t$ described above is a Gibbs-Markov flow [12, Section 6]. Therefore, the mixing rates for non-uniformly hyperbolic flows can be deduced from the corresponding results for Gibbs-Markov flows, see [12, Corollary 8.1].

For observables *v* and *w*, let $\rho _t(v,w)$ denote the decay of correlations of the geometrical neutral Lorenz flow; that is,

32 $$\begin{aligned} \rho _t(v,w)=\Bigg |\int v\cdot w\circ N^td\mu -\int vd\mu \int wd\mu \Bigg |, \end{aligned}$$

where $\mu$ is the SRB measure of ${\text {N}}^t$. Before giving the proof of Theorem 1.1 for the neutral models 1 and 2, we will give the definitions of the space of observables.

Let (*M*, *d*) be a metric space with ${\text {diam}}(M)\le 1$ and define a flow $T^t : M \rightarrow M$ on *M*. We fix $\eta \in (0,1]$ and for a given observable $v : M \rightarrow \mathbb {R}$ we define

$$\begin{aligned} |v|_{C^\eta }=\sup _{x\ne y}\frac{|v(x)-v(y)|}{d(x,y)^\eta }, \end{aligned}$$

and the norm $\left\Vert v\right\Vert _{{C^\eta }}=|v|_\infty +|v|_{C^\eta }$. We define the Banach space of Hölder functions on *M* by $C^\eta (M)$; i.e.,

$$\begin{aligned} C^\eta (M)=\{v : M \rightarrow \mathbb {R}\;|\;\left\Vert v\right\Vert _{{C^\eta }}<\infty \}. \end{aligned}$$

Furthermore, let

$$\begin{aligned} |v|_{C^{0,\eta }}=\sup _{\begin{array}{c} x\in M\\ t>0 \end{array}}\frac{|v(T^t(x))-v(x)|}{t^\eta } \end{aligned}$$

and define $\left\Vert v\right\Vert _{{C^{0,\eta }}}=|v|_\infty +|v|_{C^{0,\eta }}$. We denote the space of Hölder observables in the flow direction by

$$\begin{aligned} C^{0,\eta }(M)=\{v : M \rightarrow \mathbb {R}\;|\;\left\Vert v\right\Vert _{{C^{0,\eta }}}<\infty \}. \end{aligned}$$

We will say that an observable $w : M \rightarrow \mathbb {R}$ is **differentiable in the flow direction** if

$$\begin{aligned} \partial _tw=\lim _{t\rightarrow 0}\frac{w\circ T^t-w}{t} \end{aligned}$$

exists pointwise. Let $\left\Vert w\right\Vert _{{C^{m,\eta }}}=\sum _{k=0}^m\left\Vert \partial _t^kw\right\Vert _{{C^\eta }}$. We will denote the space of observables that are *m*-times differentiable in the flow direction by

$$\begin{aligned} C^{m,\eta }(M)=\{w : M \rightarrow \mathbb {R}\;|\;\left\Vert w\right\Vert _{{C^{m,\eta }}}<\infty \}. \end{aligned}$$

For a Borel set $X\subset M$ we define $C^\eta (X)$ as above by using the restriction of the metric *d* to *X*.

### Proof Theorem 1.1

We first note that the suspension flow $F^t$ projects to a quotient suspension semiflow ${\tilde{F}}^t : {\tilde{X}}^R \rightarrow {\tilde{X}}^R$, where ${\tilde{X}}$ is the quotient space obtained from *X* after quotienting out the stable leaves and ${\tilde{F}} : {\tilde{X}} \rightarrow {\tilde{X}}$ is a Gibbs-Markov map. Proposition 3.9 ensures that the following inequality holds.

33 $$\begin{aligned} |\varphi (x)-\varphi (y)|\le C\gamma ^{s(x,y)}\inf _{Q_i}\varphi \quad \text {for all }x,\; y\in {\tilde{Q}}_i,\; i\ge 1, \end{aligned}$$

where $\{{\tilde{Q}}_i\}_{i\ge 0}$ is a countable Lebesgue modulo zero partition into subintervals, $0<\gamma <1$ and *s*(*x*, *y*) is the separation time.

Therefore, we have that ${\tilde{F}}^t$ is a Gibbs-Markov semiflow and consequently that $F^t$ is a Gibbs-Markov flow. Then the conclusion follows from [12, Corollary 8.1].

There are still four details concerning the hypothesis in [12, Corollary 8.1] that we have not mentioned yet. The first one is regarding condition (H); for us this condition holds automatically since *R* is constant along stable leaves.

The second concerns the absence of approximate eigenfunctions for $F^t$. Melbourne gave in [30, Chapter 5] sufficient conditions for the absence of approximate eigenfunctions, namely the existence of a finite subsystem with positive lower box dimension. Hence, it follows from Proposition 3.12 and Lemma 8.9 in [12] that $F^t$ has absence of approximate eigenfunctions.

The third concerns the tails of *R*; that is, we want to estimate $\mu _X(R>t)$. From Observation 3.5 we know that $\mu _X(\tau >n)$ has exponential tails; i.e., there exists a constant $c_0>0$ such that $\mu _X(\tau >n)=\mathcal {O}({\text {e}}^{-c_0n})$. Moreover, by Theorem 2.1 we have that $\mu _X(r_{{\text {Neu}}}>t)$ has polynomial tails; that is, there is a constant $c_1>0$ such that $\mu _X(r_{{\text {Neu}}}>t)=\mathcal {O}(c_1t^{-\beta _2})$, where $\beta _2=\frac{a_2+b_2}{2b_2}$. Then by [18, Proposition 5.1] we have that $\mu _X(R>t)=\mathcal {O}((\ln t)^{\beta _2}t^{-\beta _2})$.

The fourth and last detail concerns how to improve the estimates for $\mu _X(R>t)$ and remove the logarithmic term. For this, we make use of [11, Lemma 4.1]. There the settings is made for infinite horizon planar periodic Lorentz gases, for that setting the tails of the flow time (in this work denoted by $r_{{\text {Neu}}}$) is of order $\mathcal {O}(t^{-2})$. By replacing in [11, Lemma 4.1] the order of the tails from $\mathcal {O}(t^{-2})$ to $\mathcal {O}(c_1t^{-\beta _2})$ we can use the same proof to remove the logarithmic term. Hence, we have that $\mu _X(R>t)=\mathcal {O}(t^{-\beta _2})$. With this we conclude our proof. $\square$

The natural questions is about the lower bounds, i.e., if the bounds given in this theorem are sharp.

Although we definitely think they are, the currently available literature is insufficient to conclude this, although the margin is fairly narrow.

In [24], the renewal operator methods are developed to get such lower bounds, but his paper is for maps, not flows. Melbourne and Terhesiu come the closest in [31], where they consider suspension semiflows with polynomial roof functions over Gibbs-Markov base maps, and indeed, their results imply polynomial mixing for the flow $F^t : X^R \rightarrow X^R$, for Hölder observables that are constant on the stable fibers. The step from this suspension flow to the actual flow ${\text {N}}^t : \Lambda _{{\text {Neu}}} \rightarrow \Lambda _{{\text {Neu}}}$, however, is not trivial at all. (Here the effect of the second inducing step needs to be undone, in a way).

In [18] this step was taken for discrete suspensions (i.e., Young towers), specifically for billiard maps, but not for flows. However, the lower bounds that we obtain for $F^t : X^R \rightarrow X^R$ as a corollary of the results in [31] are sufficient to prove stable laws (with exponent $1/\beta _2 \in (1,2]$) for the neutral Lorenz flow ${\text {N}}^t : \Lambda _{{\text {Neu}}} \rightarrow \Lambda _{{\text {Neu}}}$, cf. [15, 19].

## Numerical Analysis and Results

In this section we provide the results of the numerical approximation we obtained for the exponent $\beta$ of the Dulac map (see Equation (8)) and the exponent $\beta _2$ of the tails of the return map (see Theorem 2.1) for the two-dimensional setting (the setting of [17]) and for the 3-dimensional setting concerning this work.

### Numerics of the 2-Dimensional Case

To verify the existing theoretical asymptotics from [17], we will start the numerical analysis in the 2-dimensional case; that is, we consider the framework of [15] and [17]. There, the following neutral form was considered:

34 $$\begin{aligned} \left( \begin{array}{ccc} {\dot{x}}\\ {\dot{y}}\\ \end{array}\right) = \left( \begin{array}{ccc} x(a_0x^\kappa + a_2z^\kappa )\\ -y(b_0x^\kappa + b_2y^\kappa )\\ \end{array}\right) +\mathcal {O}(4), \end{aligned}$$

where $a_0,\;a_2,\;b_0$ and $b_2>0$ and $\Delta :=a_2b_0-a_0b_2\ne 0$. For simplicity we let $\kappa =2$. For the analysis of the Dulac map close to the neutral equilibrium of Equation (34), we take an unstable leaf $W^u(0,y_0)$ and a stable leaf $W^s(x_0,0)$, then the Dulac map $D : W^u(0,y_0) \rightarrow W^s(x_0,0)$, shown in Fig. 3, assigns the firs intersection of the integral curve through $(x,y_0)$ with the stable leaf $W^s(x_0,0)$, where $x\in W^u(0,y_0)$ and *T* is the flow time; that is, the exit time.

For the setting considered in this work, we will perform the numerical experiments with $x_0=1$. In order to corroborate the estimates of the Dulac map given by Equation (8), we expect the numerical experiments to show us that

35 $$\begin{aligned} \beta \approx \frac{\ln (y)}{\ln (x)}. \end{aligned}$$

We actually will see that

36 $$\begin{aligned} \beta =\frac{\ln (y)}{\ln (x)}-\frac{\ln (c(y_0))}{\ln (x)}+\mathcal {O}\Big (\frac{1}{\ln (x)}\Big ). \end{aligned}$$

From [15] and [17] we know that the constants $c(y_0)$ are given by a specific formula which is not easy to compute. For this reason, we decided to use the least-squares method to calculate these constants.

For the numerical experiments we will take different values of $\beta$ and 250 points $x\in [1.0 \times 10^{-5},1.0 \times 10^{-4}]$ at the unstable leave $W^u(0,y_0)$ with $y_0=1.0$. The integration method we will use for the numerical experiments concerning this work is the so-called Radau quadrature method, to deal with the numerical complications of integrating near a neutral stationary point, see [26].

**Fig. 8:**
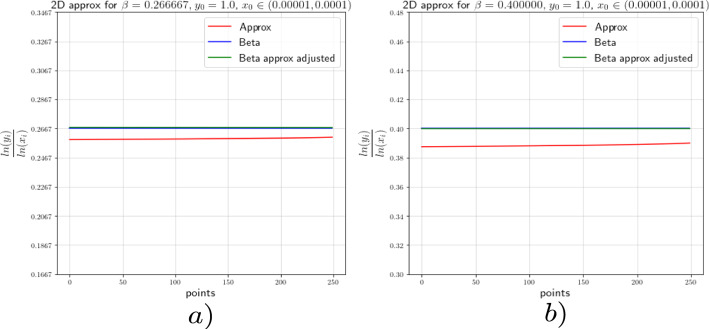
2-dimensional $$\beta $$ approximation

Figure 8*a*), *b*) show us the approximation of $\beta$ (the red graph), the adjusted approximation of $\beta$ (green graph), and the theoretical value of $\beta$ (blue graph) for $\beta =0.266$ and $\beta =0.40$, respectively. The approximation of beta is done by taking the last *y* value of each integral curve and divide it by the *x* value ranging in $[1.0 \times 10^{-5},1.0 \times 10^{-4}]$, the adjusted beta is calculated using Equation (36). The points-axis corresponds to the 250 *x* values we considered starting from $1.0 \times 10^{-4}$ and ending in $1.0 \times 10^{-5}$; that is, point 0 corresponds to the value $x=1.0 \times 10^{-4}$ and point 250 corresponds to the value $x=1.0 \times 10^{-5}$. The constants $c(y_0)=\ln (0.8)$ and $c(y_0)=1.2\ln (1.1)$ for the adjusted approximation correspond to Fig. 8*a*) and *b*), respectively. The approximations show an error that tends to decrease as we get closer to $x=0$ as depicted in the graphs. The value $\beta =0.40$ and $\beta =0.266$ are obtained by taking $a_0=15.0$, $a_2=5.0$, $b_0=1.0$ and $b_2=3.0$ and $a_0=15.0$, $a_2=6.0$, $b_0=1.0$ and $b_2=2.0$, respectively, in the vector field from Equation (34).

We proceed in the same way to perform the numerical analysis for the exponent $\beta _2$. From the estimates obtained in [17], we can see that,

37 $$\begin{aligned} \beta _2\approx -\frac{\ln (x)}{\ln (t)}, \end{aligned}$$

where *t* is the flow time that it takes a point from the unstable leaf to hit the stable leaf and $x\in [1.0 \times 10^{-5},1.0 \times 10^{-4}]$. We will actually show that

38 $$\begin{aligned} \beta _2=\frac{\ln (c(y_0))}{\ln (t)}-\frac{\ln (x)}{\ln (t)}+\mathcal {O}\Big (\frac{1}{\ln (t)}\Big ). \end{aligned}$$

**Fig. 9:**
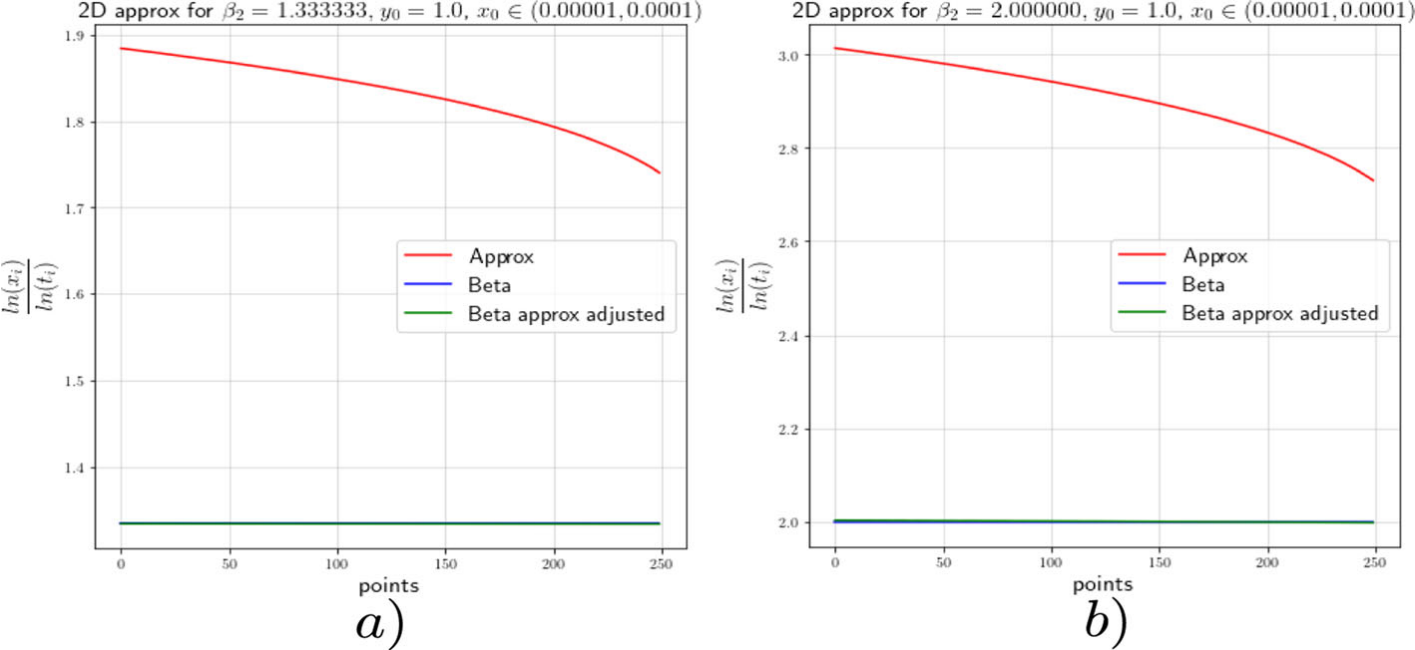
2-dimensional $$\beta _2$$ approximation

Figure 9 shows the approximation of the exponent of the tail of the return map with values $\beta _2=0.1333$ and $\beta _2=2.0$ which correspond to the values $\beta =0.400$ and $\beta =0.266$, respectively. For this case the constants for the adjusted approximations are $c(y_0)=\ln (0.06)$ and $c(y_0)=\ln (0.045)$ for Fig. 9*a*) and *b*), respectively. From the approximations we see, as before, that the error decreases as we approach to $x=0$.

### Numerics of the 3-Dimensional Case

In this subsection we will perform the numeric experiments for the 3-dimensional models. We will start with Neutral model 1. Recall that the Neutral model 1 was given by Equation (39)

39 $$\begin{aligned} \left( \begin{array}{ccc} {\dot{x}}\\ {\dot{y}}\\ {\dot{z}}\\ \end{array}\right) = N\left( \begin{array}{ccc} x\\ y\\ z\\ \end{array}\right) = \left( \begin{array}{ccc} x(a_0x^2+a_1y^2+a_2z^2)\\ -\ell y\\ -z(b_0x^2+b_1y^2+b_2z^2)\\ \end{array}\right) +\mathcal {O}(4), \end{aligned}$$

where $a_0,\;a_1,\;a_2,\;b_0,\;b_1,\;b_2$ and $\ell >0$ and $\Delta :=a_2b_0-a_0b_2\ne 0$.Precise asymptotics are not available, but since *y*(*t*) decreases exponentially fast, the same asymptotics as in (34) are expected, and our numerics indeed confirm this. Note that the strong stable direction of (39) is still purely *y*-directed.

For the analysis of the Dulac map close to the neutral equilibrium of Equation (39), we will perform the numerical analysis on $N_1 : \Sigma \rightarrow S$. For the purpose of this work, we want to show with the numeric experiment that the *x* and *z* components behave like the 2-dimensional model from the previous subsection regardless of the *y* value. To perform the numerical analysis we will take different unstable leaves $W^u(x,y_0,z_0)$ and a stable leaf $W^s(1,0,0)$, where $y_0=1.0$ and $z_0=1.0$. Hence, like in the 2-dimensional analysis we expect the numerical experiments to show us that,

40 $$\begin{aligned} \beta \approx \frac{\ln (z)}{\ln (x)}, \end{aligned}$$

where *z* is the last value of the integral curve with initial condition $(x,y_0,z_0)$ for $x\in [1.0 \times 10^{-5},1.0 \times 10^{-4}]$. Again, we will actually show that

41 $$\begin{aligned} \beta =\frac{\ln (z)}{\ln (x)}-\frac{\ln (c(z_0))}{\ln (x)}+\mathcal {O}\Big (\frac{1}{\ln (x)}\Big ). \end{aligned}$$

**Fig. 10:**
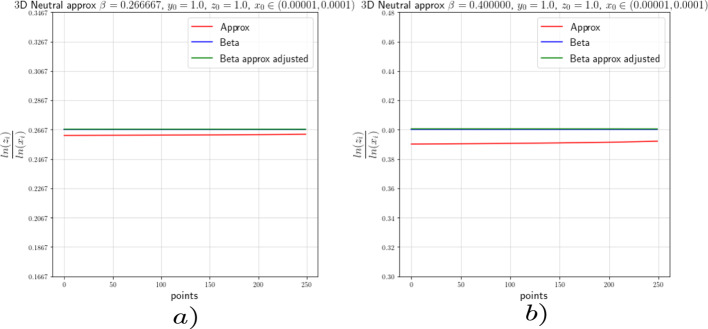
Neutral model 1 $\beta$ approximation

As before, we will use the Radau quadrature method and take 250 points for the values of *x* starting from $1.0 \times 10^{-4}$ and ending with $1.0 \times 10^{-5}$; that is, point 0 and point 250 correspond to $x=1.0 \times 10^{-4}$ and $x=1.0 \times 10^{-5}$, respectively. We will consider the same values of $\beta$ we considered in the previous subsection. The approximation of $\beta$, corresponding to the red line in all figures, is done by taking the last *z* value of each integral curve with initial condition $(x,y_0,z_0)$ and divide it by the *x* value ranging in $[1.0 \times 10^{-5},1.0 \times 10^{-4}]$, the adjusted $\beta$, plotted in green in all figures, is calculated by using Equation (41), and the theoretical value of $\beta$, corresponding to the blue graph in all figures, is obtained from the parameters $a_0,\; a_2,\;b_0$ and $b_2$ as before. The constants $c(z_0)$ were calculated using the least squares method. The constants $c(z_0)=\ln (1.1)$ and $c(z_0)=\ln (1.06)$ correspond to Fig. 10*a*) and *b*), respectively.

Next, we consider the Neutral model 2 given by Equation (42), with the parameters satisfying the usual constraints, and present the numerical results obtained by performing the same experiments we did for the Neutral model 1. We consider this form since it is no longer a skew product like the previous model and poses a new challenge to deduce its asymptotics and u decay of correlations.

42 $$\begin{aligned} \left( \begin{array}{ccc} {\dot{x}}\\ {\dot{y}}\\ {\dot{z}}\\ \end{array}\right) = G\left( \begin{array}{ccc} x\\ y\\ z\\ \end{array}\right) = \left( \begin{array}{ccc} x(a_0x^2+a_2z^2)\\ -\ell y(1+c_0x^2+c_2z^2)\\ -z(b_0x^2+b_2z^2)\\ \end{array}\right) +\mathcal {O}(4). \end{aligned}$$

**Fig. 11:**
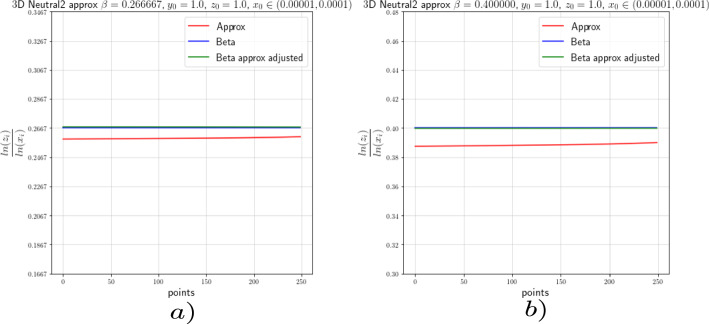
Neutral model 2 $\beta$ approximation

Figure 11*a*) and *b*) show us the the numerical approximations of the Neutral model 2 for $\beta =0.40$ and $\beta =0.266$ with constants $c(z_0)=\ln (1.2)$ and $c(z_0)=\ln (1.08)$, respectively. We observe that the constants $c(z_0)$ and $c(y_0)$ from Equation (41) and Equation (36), respectively, are almost equal; that is, the *x* and *z* components of the 3-dimensional model behaves like the *x* and *y* component of the 2-dimensional model.

Until now we have performed the numerical experiments for the Neutral model 1 and 2 corresponding to the normal forms given by Equations (39) and (42), respectively. We saw there that for both models, the asymptotic behaviour of the *x* and *z* components are the same as the *x* and *y* component of the 2-dimensional model. Recall that for this two models we had explicit formulas for the map $N : \Sigma \rightarrow S$ and hence for the modified Poincaré map $P_{{\text {Neu}}} : \Sigma \rightarrow \Sigma$. Our goal is to see whether the asymptotic behaviour of the Neutral model 3 given by Equation (4) is similar to the other two models. Therefore, the numerical results obtained for the Neutral models 1 and 2 will be our reference and we will compare them to the numerical results obtained for the third model.

**Fig. 12:**
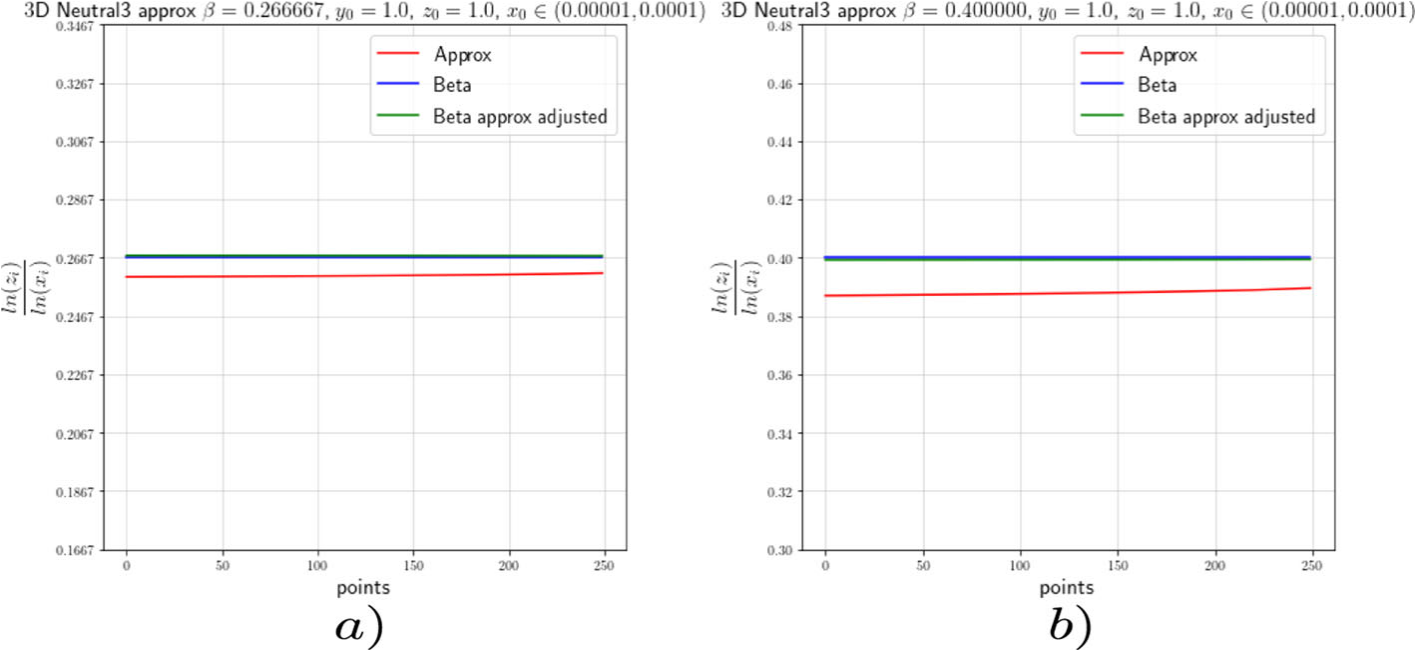
Neutral model 2 $\beta$ approximation

Figure 12*a*) and *b*) show us the the numerical approximations of the Neutral model 2 for $\beta =0.40$ and $\beta =0.266$ with constants $c(z_0)=\ln (1.12)$ and $c(z_0)=\ln (1.07)$, respectively. We observe again that the *x* and *z* components of the 3-dimensional model behaves like the *x* and *y* component of the 2-dimensional model. From this numerical experiments we can conclude that the behaviour of the map $N_1$ obtained by considering the neutral model 3 is asymptotically similar to the other two models; that is, the asymptotics of the Dulac map of the three models are similar.

### Numerics of the Tails of the Return Map in the 3-Dimensional Case

Next we will perform the numeric experiments for the 3-dimensional models and see the approximations for the exponent of the decay of correlations; that is, for the exponent $\beta _2$. The general Neutral model or neutral model 3 is given by Equation (4), where $a_0,\;a_1,\;a_2,\;b_0,\;b_1,\;b_2,\;c_0,\;c_2$ and $\ell >0$ and $\Delta :=a_2b_0-a_0b_2\ne 0$. Note that the neutral model 1 and the neutral model 2 are obtained from the general neutral model if we let $c_0,\;c_2=0$ and if we let $a_1,\;b_1=0$, respectively.

In the previous subsection we saw the numerical analysis on $N_1 : \Sigma \rightarrow S$ and showed, with the numeric experimentation, that the *x* and *z* components behaves like the 2-dimensional model. For the next numerical analysis, we will take an unstable leaf $W^u(x,y_0,z_0)$ and a stable leaf $W^s(1,0,0)$, where $y_0=1.0$ and $z_0=1.0$. From the estimates obtained in [17] we can see that

43 $$\begin{aligned} \beta _2\approx -\frac{\ln (x)}{\ln (t)}, \end{aligned}$$

where *t* is the flow time that it takes a point from the unstable leaf to hit the stable leaf and $x\in [1.0 \times 10^{-5},1.0 \times 10^{-4}]$. We will actually show that

44 $$\begin{aligned} \beta _2=\frac{\ln (c(z_0))}{\ln (t)}-\frac{\ln (x)}{\ln (t)}+\mathcal {O}\Big (\frac{1}{\ln (t)}\Big ). \end{aligned}$$

As before, we will use the Radau quadrature method and take 50 points for the values of *x* starting from $1.0 \times 10^{-4}$ and ending with $1.0 \times 10^{-5}$; that is, point 0 and point 50 correspond to $x=1.0 \times 10^{-4}$ and $x=1.0 \times 10^{-5}$, respectively. We will consider the same values of $\beta$ we considered in the previous subsection. The approximation of $\beta _2$, corresponding to the red line in all figures, is done by taking the *x* value, ranging in $[1.0 \times 10^{-5},1.0 \times 10^{-4}]$, of each integral curve with initial condition $(x,y_0,z_0)$ and divide it by the flow time *t*, the adjusted approximation of $\beta _2$, shown in green in all figures, is calculated by using Equation (44), and the theoretical value of $\beta _2$, corresponding to the blue graph in all figures, is obtained from the parameters $a_2$ and $b_2$; that is, $\beta _2=\frac{a_2+b_2}{2b_2}$.

**Fig. 13:**
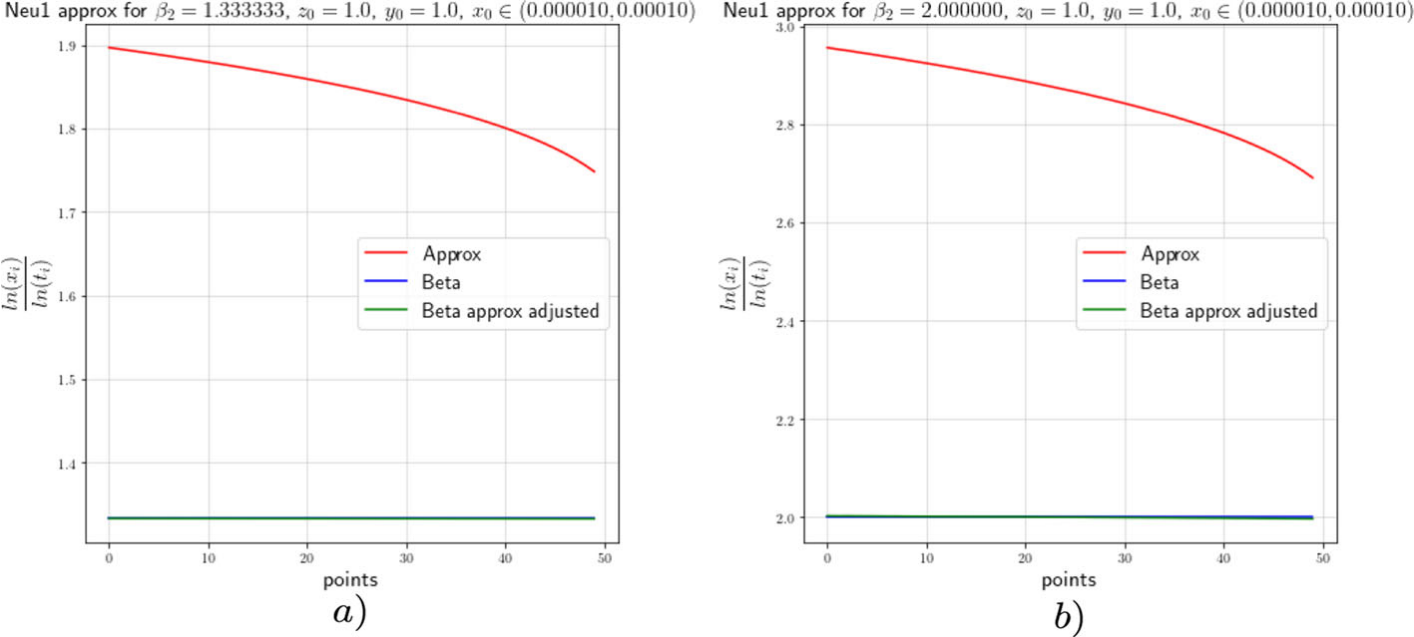
Neutral model 2 $\beta$ approximation

We start considering the neutral model 1. Figure 13*a*) shows the approximation for $\beta _2=1.333$ which corresponds to the case $\beta =0.40$, for the adjusted approximation the constant is $\ln (c(z_0))=0.06$ and *b*) displays the approximation for $\beta _2=2.0$ corresponding to the case $\beta =0.266$ with adjustment constant $\ln (c(z_0))=0.05$.

**Fig. 14:**
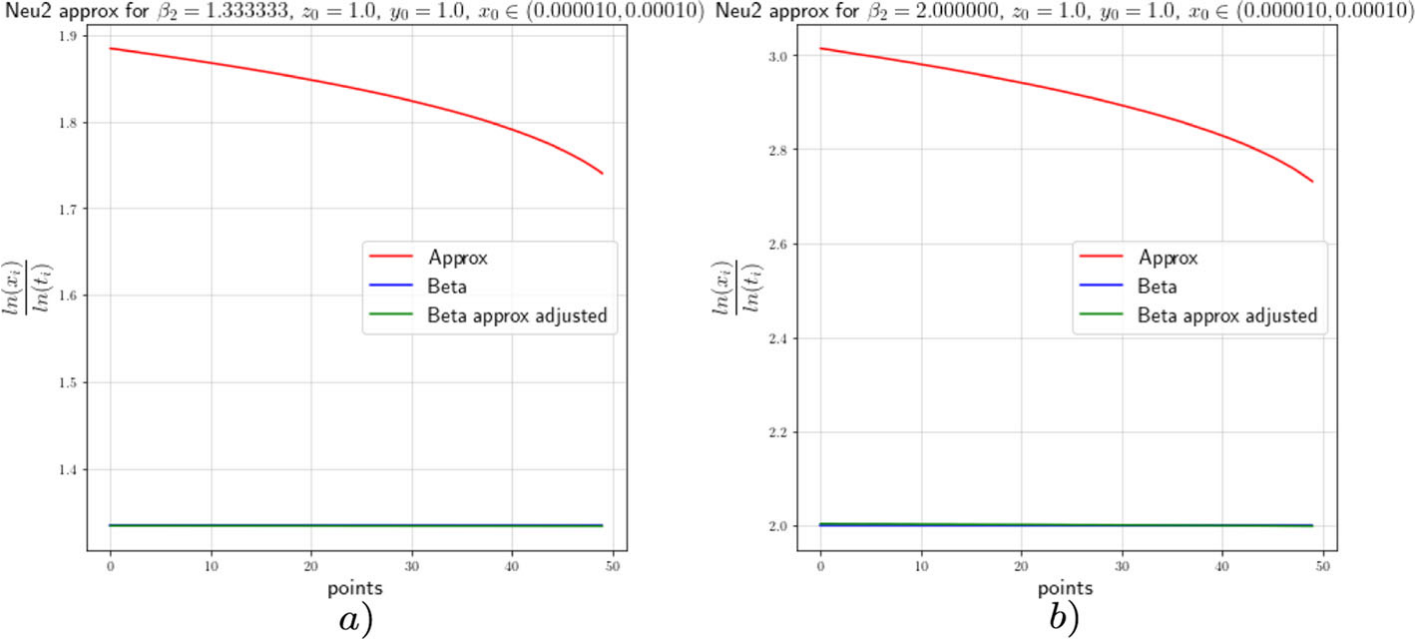
Neutral model 2 $\beta$ approximation

Next, we consider the neutral model 2. Figure 14*a*) and *b*) show the approximation for $\beta _2=1.333$ and $\beta _2=2.0$, respectively. Their adjusted approximation the constant are $\ln (c(z_0))=0.06$ and $\ln (c(z_0))=0.04$, respectively.

**Fig. 15:**
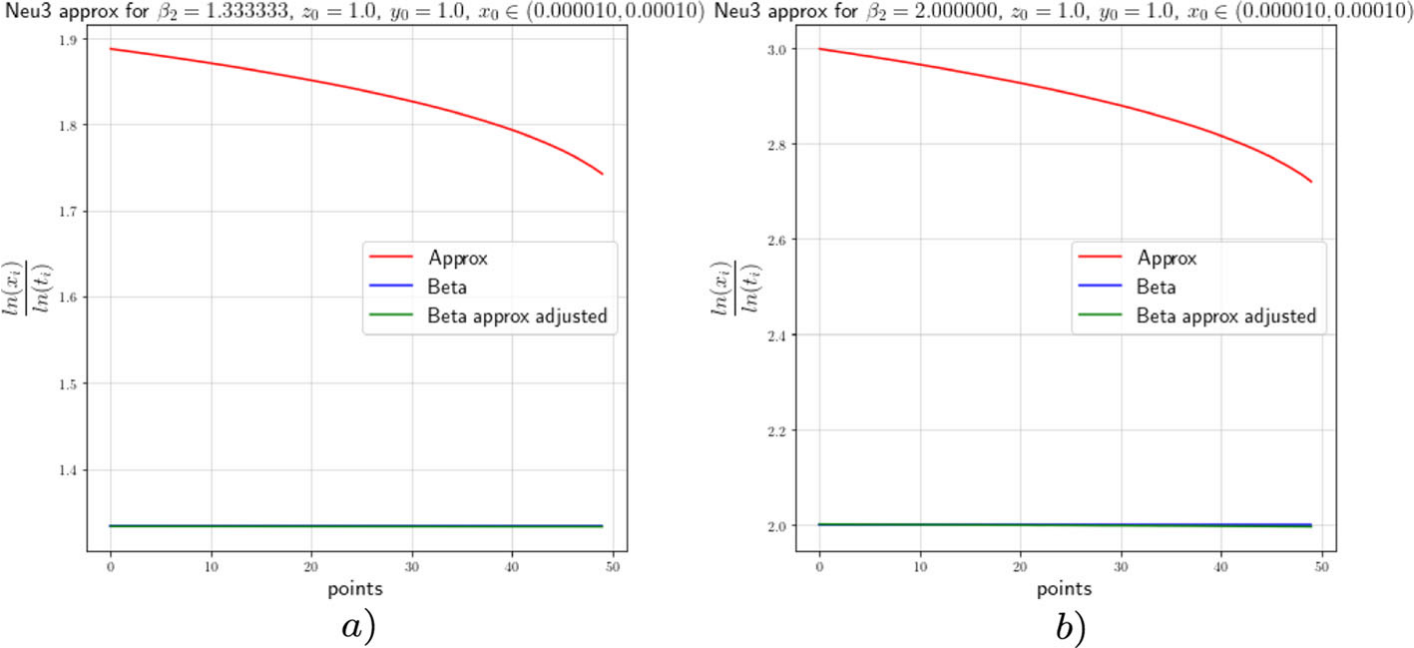
Neutral model 2 $\beta$ approximation

Finally, we consider the neutral model 3. Figure 15*a*) and *b*) show the approximation for $\beta _2=1.333$ and $\beta _2=2.0$, respectively. Their adjusted approximation the constant are $\ln (c(z_0))=0.06$ and $\ln (c(z_0))=0.04$, respectively.

This numerical experiment has shown us a good approximation of the exponent of the decay of correlations for the 3 neutral models. From this we can deduce the same results concerning the decay of correlations, and obtaining Theorem 1.1 in its full generality.

## Data Availability

The methods of how the numerical graphics were computed are given in the paper. For further details on the hardware and software specifications, as well as the code implemented to produce the graphical results, please see the arXiv version [14].

## Footnotes

1 In [17], the measure of interest is the SBR measure, but because the SBR measure is flow-invariant (hence, with smooth holonomies in the flow-direction) and conditioned to unstable leaves is absolutely continuous w.r.t. Lebesgue (in fact, equivalent to Lebesgue with a density that is bounded and bounded away from 0, see [17, Proposition 3.1] and [28, Lemma 4.1]) and the separate unstable leaves are $C^1$ smooth curves, it is justified to work with the Lebesgue measure on $W^u(0,z_0)$.

2 Nondegenerate here is meant for the derivative of the one-dimensional map $f_{{\text {Neu}}}$ and shouldn’t be confused with the degeneracy at the singularity 0 concerning the eigenvalues being zero.
